# Forgetting in Reinforcement Learning Links Sustained Dopamine Signals to Motivation

**DOI:** 10.1371/journal.pcbi.1005145

**Published:** 2016-10-13

**Authors:** Ayaka Kato, Kenji Morita

**Affiliations:** 1 Department of Biological Sciences, Graduate School of Science, The University of Tokyo, Tokyo, Japan; 2 Physical and Health Education, Graduate School of Education, The University of Tokyo, Tokyo, Japan; École Normale Supérieure, College de France, CNRS, FRANCE

## Abstract

It has been suggested that dopamine (DA) represents reward-prediction-error (RPE) defined in reinforcement learning and therefore DA responds to unpredicted but not predicted reward. However, recent studies have found DA response sustained towards predictable reward in tasks involving self-paced behavior, and suggested that this response represents a motivational signal. We have previously shown that RPE can sustain if there is decay/forgetting of learned-values, which can be implemented as decay of synaptic strengths storing learned-values. This account, however, did not explain the suggested link between tonic/sustained DA and motivation. In the present work, we explored the motivational effects of the value-decay in self-paced approach behavior, modeled as a series of ‘Go’ or ‘No-Go’ selections towards a goal. Through simulations, we found that the value-decay can enhance motivation, specifically, facilitate fast goal-reaching, albeit counterintuitively. Mathematical analyses revealed that underlying potential mechanisms are twofold: (1) decay-induced sustained RPE creates a gradient of ‘Go’ values towards a goal, and (2) value-contrasts between ‘Go’ and ‘No-Go’ are generated because while chosen values are continually updated, unchosen values simply decay. Our model provides potential explanations for the key experimental findings that suggest DA's roles in motivation: (i) slowdown of behavior by post-training blockade of DA signaling, (ii) observations that DA blockade severely impairs effortful actions to obtain rewards while largely sparing seeking of easily obtainable rewards, and (iii) relationships between the reward amount, the level of motivation reflected in the speed of behavior, and the average level of DA. These results indicate that reinforcement learning with value-decay, or forgetting, provides a parsimonious mechanistic account for the DA's roles in value-learning and motivation. Our results also suggest that when biological systems for value-learning are active even though learning has apparently converged, the systems might be in a state of dynamic equilibrium, where learning and forgetting are balanced.

## Author Summary

Dopamine (DA) has been suggested to have two reward-related roles: (1) representing reward-prediction-error (RPE), and (2) providing motivational drive. Role(1) is based on the physiological results that DA responds to unpredicted but not predicted reward, whereas role(2) is supported by the pharmacological results that blockade of DA signaling causes motivational impairments such as slowdown of self-paced behavior. So far, these two roles are considered to be played by two different temporal patterns of DA signals: role(1) by phasic signals and role(2) by tonic/sustained signals. However, recent studies have found sustained DA signals with features indicative of both roles (1) and (2), complicating this picture. Meanwhile, whereas synaptic/circuit mechanisms for role(1), i.e., how RPE is calculated in the upstream of DA neurons and how RPE-dependent update of learned-values occurs through DA-dependent synaptic plasticity, have now become clarified, mechanisms for role(2) remain unclear. In this work, we modeled self-paced behavior by a series of ‘Go’ or ‘No-Go’ selections in the framework of reinforcement-learning assuming DA's role(1), and demonstrated that incorporation of decay/forgetting of learned-values, which is presumably implemented as decay of synaptic strengths storing learned-values, provides a potential unified mechanistic account for the DA's two roles, together with its various temporal patterns.

## Introduction

Electrophysiological [1] and fast-scan cyclic voltammetry (FSCV) [2, 3] studies have conventionally shown that dopamine (DA) neuronal activity and transmitter release respond to unpredicted but not predicted reward, consistent with the suggestion that DA represents reward-prediction-error (RPE) [1, 4]. On the other hand, recent FSCV studies [5–8] have found DA response sustained towards presumably predictable reward, arguing that it may represent sustained motivational drive. DA's roles in motivation processes have long been suggested [9–13] primarily from pharmacological results. A key finding is that post-training blockade of DA signaling causes motivational impairments such as slowdown of behavior (e.g., [14]), and this is difficult to explain with respect to the known role of DA in RPE representation because post-training RPE should be negligible so that blockade of RPE should have little impact.

Therefore it has been considered that DA has two distinct reward-related roles, (1) representing RPE and (2) providing motivational drive, and these are played by phasic and tonic/sustained DA, respectively. Normative theories have been proposed for both the role as RPE [4] and the role as motivational drive [15, 16] in the framework of reinforcement learning (RL). On the other hand, as for the underlying synaptic/circuit mechanisms, much progress has been made for the role as RPE but not for the role as motivational drive. Specifically, how RPE is calculated in the upstream of DA neurons and how released DA implements RPE-dependent update of state/action values through synaptic plasticity have now become clarified [17–20]. In contrast, both the upstream and downstream mechanisms for DA's motivational role remain more elusive.

In fact, FSCV studies that found sustained DA signals [5, 8] have shown that those DA signals exhibited features indicative of RPE. Moreover, sustained response towards presumably predictable reward has also been found in the activity of DA neurons [21, 22], and these studies have also argued that the DA activity represents RPE. Consistent with these views, we have recently shown [23] that RPE can actually sustain after training if decay/forgetting of learned values, which can presumably be implemented as decay of plastic changes of synaptic strengths, is assumed in RL. It was further indicated that whether RPE/DA sustains or not can be coherently understood as reflecting differences in how fast learned values decay in time: faster decay causes more sustained RPE/DA. However, this account did not explain the suggested link between sustained DA and motivation. Even on the contrary, decay of learned values is apparently wasteful and could be perceived as a loss of motivational drive.

In several recent studies reporting sustained DA signals [5–8], a common feature is that self-paced actions are required, as argued in [8]. We conjectured that this feature could be critical for the putative motivational functions of sustained DA signals. However, in our previous study [23], such a feature was not incorporated: our previous model was extremely simple and assumed that the subject automatically moved to the next state at every time step. In the present work, we constructed a new model, which incorporated the requirement of self-paced approach towards a goal, represented as a series of ‘Go’ or ‘No-Go’ (or ‘Stay’) selections, into RL with decay of learned values. Using this new model, we investigated: (1) if the model (as well as the previous non-self-paced model) generates both phasic and sustained RPE/DA signals so that their mechanisms can be coherently understood, (2) if the model demonstrates any association between sustained DA signals and motivation, and (3) if the model can mechanistically account for the key experimental findings that suggest DA's roles in motivation, specifically, the (i) slowdown of self-paced behavior by post-training blockade of DA signaling [14], (ii) severe impairment of effortful actions to obtain rewards, but not of seeking of easily obtainable rewards, by DA blockade [11, 24], and (iii) relationships between the reward amount, the level of motivation reflected in the speed of behavior, and the average level of DA [7]. Through simulations and mathematical (bifurcation) analyses, we have successfully answered these questions.

## Results

### The value-decay facilitates fast goal-reaching, and reproduces the slowdown caused by DA blockade

**Fig 1:**
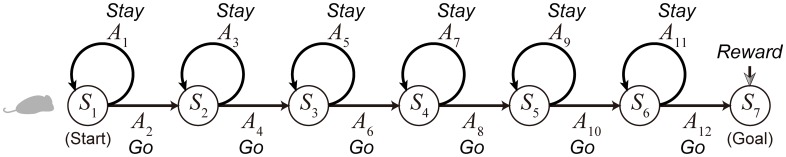
Modeling the behavior of subject performing a task that requires self-paced voluntary approach (whether spatially or not) towards a goal. We posited that self-paced voluntary approach can be represented as a series of ‘Go’ or ‘Stay’ selections, as illustrated here. Subject starts from *S*_1_, and chooses ‘Go’ or ‘Stay’ according to their learned values in a soft-max manner in each state until reaching the goal (*S*_7_), where reward is obtained. The values of actions (‘Go’ and ‘Stay’) are learned through temporal-difference (TD) reinforcement learning (RL) incorporating the decay of learned values (referred to as the ‘value-decay’): learned value of arbitrary action (‘Go’ or ‘Stay’) is multiplied, at every time step, by (1 –*φ*), where *φ* (0 ≤ *φ* ≤ 1) represents the decay rate: *φ* = 0 corresponds to the case without value-decay.

We modeled a behavioral task requiring self-paced voluntary approach (whether spatially or not) towards a goal as a series of ‘Go’ or ‘Stay’ (‘No-Go’) selections as illustrated in Fig 1. We then simulated subject's behavior by a temporal-difference (TD) RL model incorporating the decay of learned values (referred to as the ‘value-decay’ below). Specifically, we assumed that at every time step the subject selects ‘Go’ or ‘Stay’ depending on their learned values, which are updated according to RPE (TD error) when the corresponding action is taken. In addition, we also assumed that the learned values of all the actions (whether selected or not) decay in time at a constant rate (see the Materials and Methods for details). RPE at each time step was assumed to be represented by the level of DA at the time step, and the value decay was assumed to be implemented as a decay of plastic changes of synaptic strengths storing learned values.

**Fig 2:**
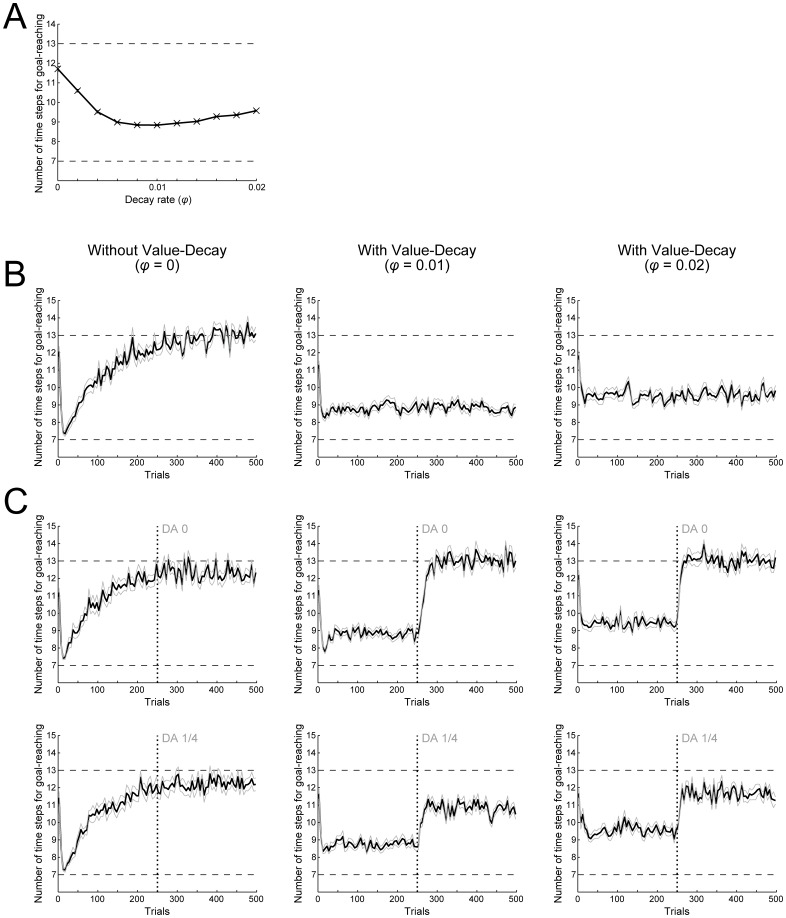
RL model with the value-decay achieves fast goal-reaching, and reproduces the slowdown caused by post-training blockade of DA signaling. **(A)** Number of time steps needed for goal-reaching averaged over 500 trials (vertical axis) in the cases with various rates of value-decay (horizontal axis). The rate of the value-decay is referred to as the decay rate and represented by the parameter *φ*: "decay rate *φ =* 0" corresponds to the case without value-decay. The error bar indicates the mean ± standard error (SE) of 20 simulations. The bottom dashed line indicates the theoretical minimum number of time steps needed for goal-reaching (including the steps at the start and the goal) and the top dashed line indicates the chance level: these are also applied to (B) and (C). **(B)** The thick black lines indicate trial-by-trial changes of the number of time steps needed for goal-reaching averaged over every 5 trials (vertical axis) along with the progression of trials (horizontal axis). The gray lines indicate the mean ± SE of 20 simulations. The left, middle, and right panels show the cases with *φ =* 0 (without the value-decay), *φ =* 0.01, and *φ =* 0.02, respectively: this is also applied to (C). **(C)** Effects of post-training blockade of DA signaling on the number of time steps needed for goal-reaching. During simulations similar to (B), the size of TD-reward-prediction-error(RPE)-dependent increment of action values was reduced to zero (top panels) or to a quarter of the original size (bottom panels) after 250 trials were completed (indicated by the vertical dotted lines). The other configurations are the same as those in (B).

Fig 2A shows the number of time-steps needed for goal-reaching (i.e., from the start to the goal in a single trial; referred to as the ‘time needed for goal-reaching’ below) averaged over 500 trials, with the rate of the value-decay (referred to as the ‘decay rate’ below) varied. As shown in the figure, the time needed for goal-reaching is minimized in the case with a certain degree of value-decay. In other words, introduction of the value-decay can facilitate fast goal-reaching. Fig 2B shows the trial-by-trial change of the time needed for goal-reaching. Without the value-decay (Fig 2B, left), the subject initially learns to reach the goal quickly, but subsequently a significant slowdown occurs. In contrast, with the value-decay (Fig 2B, middle and right), the time needed for goal-reaching is kept small, never showing slowdown. The observed facilitation of fast goal-reaching by introduction of the value-decay (Fig 2A) is thus accompanied with such a qualitative change in the long-term dynamics.

In the same simulated task using the same model, we examined how post-training blockade of DA signaling affects the subject's speed (i.e., the time needed for goal-reaching), again varying the decay rate. Specifically, with the assumption that DA represents RPE, we simulated the post-training DA blockade by reducing the size of RPE-dependent increment of action values to zero (complete blockade) or to a quarter of the original size (partial blockade) after 250 trials were completed. Fig 2C shows the results. As shown in the left panels of Fig 2C, without the value-decay, DA blockade causes little effect on the subject's speed. In contrast, in the case with the value-decay (Fig 2C, middle and right panels), the same DA blockade rapidly causes pronounced slowdown (i.e., increase in the time needed for goal-reaching).

### The value-decay leads to sustained positive RPE and a gradient of ‘Go’ values

**Fig 3:**
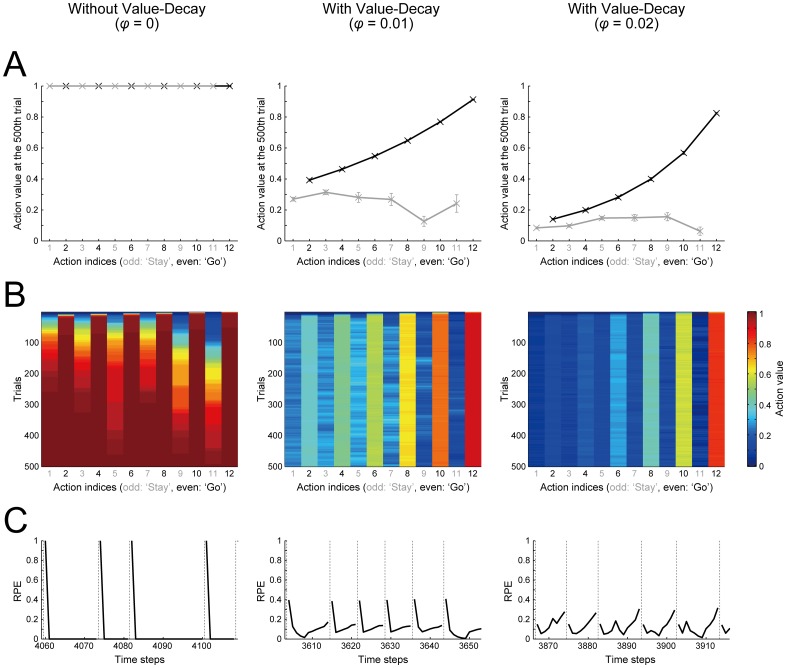
The value-decay leads to sustained RPE, which generates a gradient of ‘Go’ values towards the goal. **(A)** Action values of ‘Go’ (black lines/crosses) and ‘Stay’ (gray lines/crosses) at the end of the 500th trial. The horizontal axis indicates the indices of the actions (illustrated in Fig 1), where the odd numbers (shown in gray) indicate ‘Stay’ whereas the even numbers (black) indicate ‘Go’. The error bars show the mean ± SE of 20 simulations. The left, middle, and right panels show the cases with *φ =* 0 (without the value-decay), *φ =* 0.01, and *φ =* 0.02, respectively: this is also applied to (B) and (C). **(B)** Trial-by-trial changes of action values. The color indicates the action value averaged over 20 simulations, in reference to the rightmost color scale bar. The vertical axis indicates the trials (from the top to the bottom) and the horizontal axis indicates the indices of the actions (odd/gray: ‘Stay’, even/black: ‘Go’: Fig 1). **(C)** Examples of RPE generated in successive trials of the task. The black solid lines indicate RPE and the vertical thin dotted lines delimit individual trials.

In order to explore mechanisms underlying the fast goal-reaching achieved with the value-decay and its impairment by DA blockade, we examined the action values of ‘Go’ and ‘Stay’ at each state. The black and gray lines in Fig 3A respectively show the action values of ‘Go’ and ‘Stay’ at the end of the 500th trial, and Fig 3B shows their trial-by-trial evolutions. Without the value-decay (left panels of Fig 3A and 3B), all the action values are eventually almost saturated to the reward amount (= 1), so that there remains little difference between the action values of ‘Stay’ and ‘Go’ at any states. As a result, subject should choose ‘Stay’ as frequently as ‘Go’. This explains the observed slowdown in the case without the value-decay (Fig 2B, left panel). In contrast, with the value-decay (Fig 3A and 3B, middle and right panels), the action values of ‘Go’ shape a sustained gradient from the start to the goal, while the actions values of ‘Stay’ remain relatively small.

Why does the value-decay create such a gradient of ‘Go’ values? Fig 3C shows examples of RPE generated during the task. In the case without the value-decay (left panel), positive RPE is generated at the beginning of each trial, but RPE is mostly nearly zero in other epochs. This is what we usually expect from TD RL models after learning [4, 25]. On the contrary, in the case with the value-decay (Fig 3C, middle and right panels), RPE remains to be positive in most of the time, indicating that decrement of action values due to the value-decay is balanced with RPE-dependent increment. Such sustained positive RPE is then considered to create the start-to-goal gradient of ‘Go’ values. This is because RPE generated when taking ‘Go’ at state *S*_*i*_ (*i* = 1, …, 6) is calculated (see the Materials and Methods) as

$$\text{RPE}=\gamma \cdot \text{max}\left\{Q\left(‘\text{Stay}’\text{ at }S_{i+1}\right),\;Q\left(‘\text{Go}’\text{ at }S_{i+1}\right)\right\}-Q\left(‘\text{Go}’\text{ at }S_{i}\right),$$

(*γ*: time discount factor, satisfying 0 ≤ *γ* ≤ 1) which is not greater than *Q*(‘Go’ at *S*_*i* + 1_) − *Q*(‘Go’ at *S*_*i*_) provided *Q*(‘Stay’) ≤ *Q*(‘Go’) (this would naturally be expected), and then "0 < RPE" ensures

$$0<Q\left(‘\text{Go}’\text{ at }S_{i+1}\right)-Q\left(‘\text{Go}’\text{ at }S_{i}\right)\underset{}{\Leftrightarrow }Q\left(‘\text{Go}’\text{ at }S_{i}\right)<Q\left(‘\text{Go}’\text{ at }S_{i+1}\right),$$

which indicates a gradient towards the goal.

Looking at the pattern of RPE (Fig 3C), in the case with a relatively larger value-decay, RPE exhibits a ramp towards the goal (Fig 3C, right; notably, this decay rate does not achieve the fastest goal-reaching, but still realizes a faster goal-reaching than the case without value-decay: cf. Fig 2A). This resembles the experimentally observed ramp-like patterns of DA neuronal activity [21, 22] or striatal DA concentration [5–8] as we have previously suggested using the non-self-paced model [23]. But with a milder value-decay, RPE peaks both at the start and towards the goal, with the former more prominent (Fig 3C, middle). In this way, our model generates various patterns of RPE, from phasic to ramping, depending on the decay rate, or indeed the relative strength of the value-decay to the number of states. This could potentially be in line with the fact that the studies reporting DA ramping [5–8, 21, 22] used operant or navigation tasks in which several different states within a trial seem likely to be defined whereas the studies reporting clearly phasic DA response [1, 3] used a simple classical conditioning task where a smaller number of states might be defined.

It has been also found in other studies [5, 8] that elevations in DA levels occurred earlier in later task sessions. According to our simulation results (Fig 3C), such a change could potentially be explained in our model if the decay rate gradually decreases (i.e., from the right panel of Fig 3C to the middle panel). In our simulations, such a decrease in the decay rate is in the direction towards an optimal decay rate in terms of the time needed for goal-reaching averaged over 500 trials (Fig 2A). This suggests that the experimentally observed changes in the DA response pattern across sessions [5, 8] might be an indicative of meta-learning processes to adjust the decay rate to an optimal level. Despite these potentially successful explanations of the various DA response patterns, however, not all the patterns can be explained by our model. In particular, it has been shown that the DA concentration decreases during the reward delivery (sucrose infusion for 6 sec) [2]. Our model does not explain such a decrease of DA: to explain this, it would be necessary to extend the model to describe the actual process of reward delivery/consumption.

### Mechanistic explanations of the motivational impairments caused by DA blockade

The reason why the blockade of DA signaling causes slowdown in the cases with the value-decay but not in the cases without the value-decay in our model (Fig 2C) can also be understood by looking at RPE. Specifically, in the cases with the value-decay, positive RPE is continued to be generated at every state (Fig 3C, middle and right), and each ‘Go’ value is kept around a certain value (Fig 3B, middle and right) because increment according to RPE and decrement due to the value-decay are balanced. Then, if DA signaling is blocked and the size of RPE-dependent increment is reduced, such a balance is perturbed and thereby ‘Go’ values decrease, resulting in the slowdown. In contrast, in the cases without the value-decay, sustained positive RPE is generated only at the beginning of each trial (Fig 3C, left), and it does not increase the value of ‘Go’ taken later in the trial. Thus, after learning has settled down, ‘Go’ values are almost frozen, and therefore blockade of DA signaling has little impact on subject behavior.

**Fig 4:**
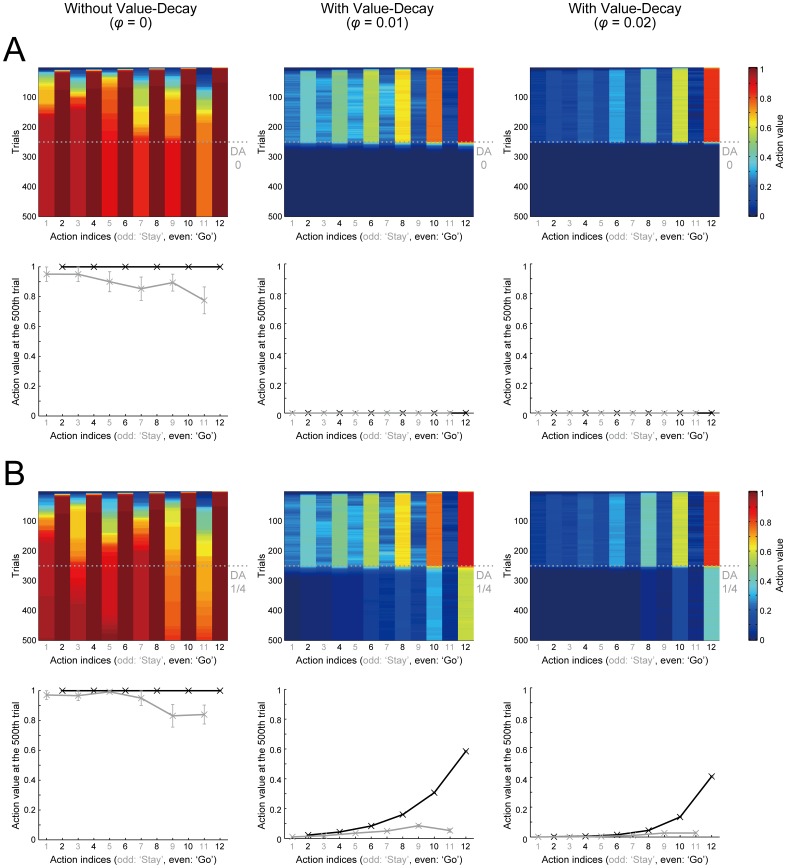
Changes in the action values caused by post-training blockade of DA signaling. The left, middle, and right panels show the cases with *φ =* 0 (without the value-decay), *φ =* 0.01, and *φ =* 0.02, respectively. The top and bottom panels of (A,B) show the trial-by-trial changes of the action values and the action values at the end of the 500th trial, respectively, in the simulations where the size of RPE-dependent increment of action values was reduced to zero (A) or to a quarter of the original size (B) after 250 trials were completed (indicated by the horizontal dotted lines). The configurations are the same as those in Fig 3B (top panels of (A,B)) or Fig 3A (bottom panels of (A,B)).

Fig 4 shows the trial-by-trial changes of the action values (the top panels of Fig 4A and 4B) and the action values at the end of the 500th trial (the bottom panels) in the simulations where the size of RPE-dependent increment of action values was reduced to zero (A) or to a quarter of the original size (B) after 250 trials were completed. As shown in these figures, the abovementioned conjectures about the effects of DA blockade on the action values were confirmed. Given that the action values are represented in the striatal neural activity, the parallel reduction in the action values and the speed for goal-reaching by DA blockade in our model can be broadly in line with a recent finding of the parallel impairment of the striatal neural representation of actions and the action vigor in DA-depleted mice [26].

Also, intriguingly, in the cases with the value-decay, after DA signaling is reduced to a quarter of the original (Fig 4B, middle and right panels), whereas the values of ‘Go’ actions distant from the goal degrade quite prominently, the values of ‘Go’ actions near the goal (i.e., *A*_12_ and *A*_10_) remain relatively large, although they are also significantly decreased from the original values. This could potentially be in line with the experimental observations that DA blockade severely impairs costly or effortful actions to obtain rewards but seeking of easily obtainable rewards are largely spared [11, 24]. In order to more directly address this issue, we simulated an experiment examining the effects of DA depletion in the nucleus accumbens in a cost-benefit decision making task in a T-maze reported in [24].

In one condition of the experiment, there was small reward in one of the two arms of the T-maze whereas there was large reward accompanied with a high cost (physical barrier) in the other arm. In the baseline period after training (exploration) of the maze, rats preferred the high-cost-high-return arm. However, DA depletion reversed the preference so that the rats switched to prefer the low-cost-low-return arm. DA depletion also increased the response latency (opening of the start door at the end of the start arm), although the latency subsequently recovered. In another condition of the experiment, the two arms contained small and large rewards as before, but neither was accompanied with a high cost. In this condition, rats preferred the large-reward arm, and DA depletion did not reverse the preference. Meanwhile, DA depletion still increased the response latency, though the latency subsequently recovered as before.

**Fig 5:**
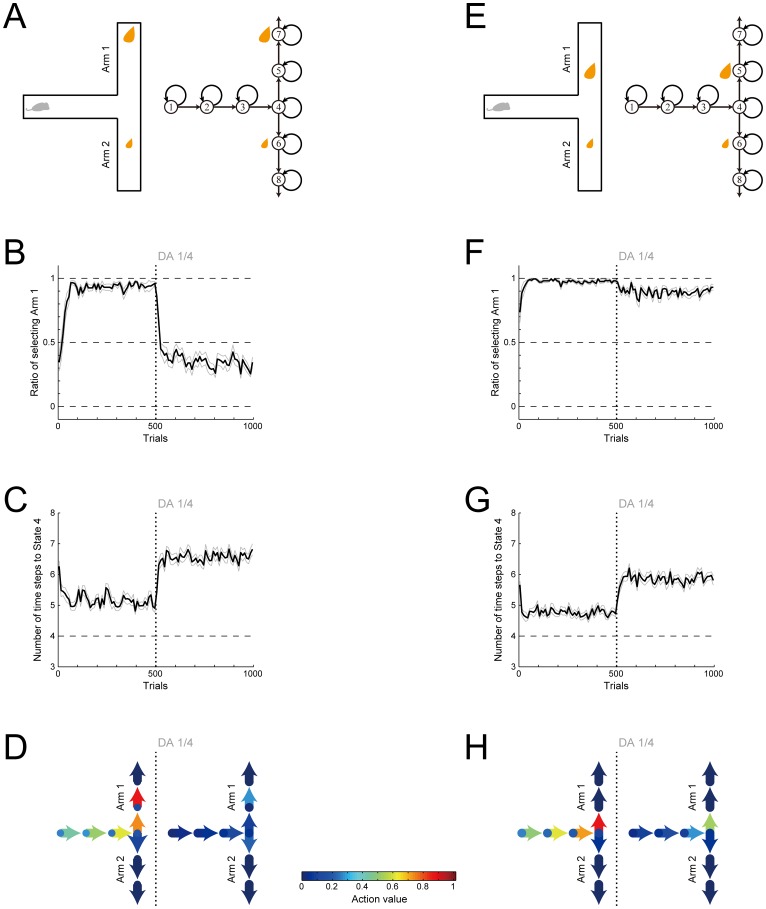
Effects of DA depletion on a cost-benefit decision making task in a T-maze simulated by the model with the value-decay. **(A)** Schematic diagram of one condition of the simulated task, in which there was small reward in one of the two arms of the T-maze (Arm 2 in the figure) whereas there was large reward accompanied with high cost, represented as an extra state preceding the reward (explicitly shown in the right panel), in the other arm (Arm 1). **(B)** Ratio of choosing the large-reward arm (Arm 1) in the simulations of the task shown in (A). The thick black line indicates the ratio of choosing Arm 1 in every 10 trials averaged over 20 simulations, and the thin gray lines indicate the mean ± SE of the 20 simulations. Post-training DA depletion was simulated in such a way that the size of RPE-dependent increment of action values was reduced to a quarter of the original size after 500 trials were completed (indicated by the vertical dotted lines). **(C)** Average number of time-steps towards the T-junction (State 4 in (A)) in the simulations of the task shown in (A). The thick black line indicates the number of time-steps averaged over every 10 trials in each of 20 simulations, and the thin gray lines indicate the mean ± SE of the 20 simulations. The bottom dashed line indicates the theoretical minimum number of time steps to State 4 (including the steps at the start and State 4). **(D)** Average action values in the simulations of the task shown in (A). The color indicates the values of actions in the T-maze (arrows: ‘Go’, circles: ‘Stay’) averaged across 251–500 trials (left, before DA depletion) or 751–1000 trials (right, after DA depletion) and averaged over 20 simulations, in reference to the bottom color scale bar. **(E)** Schematic diagram of another condition of the simulated task, in which the two arms contained small and large rewards as before, but neither was accompanied with high cost. **(F-H)** The ratio of choosing the large-reward arm (Arm 1) (F), the average number of time-steps towards the T-junction (G), and the action values (H) in the simulations of the task condition shown in (E). The configurations are the same as those in (B-D).

We simulated this experiment by representing a high cost as an extra state preceding the reward (State 5 in Fig 5A, right). Fig 5B and 5C show the ratio of choosing the large-reward arm (Arm 1 in Fig 5A) and the average time needed for reaching the T-junction (State 4 in Fig 5A, right), respectively, in the condition with a high cost in the large-reward arm (Fig 5A). Fig 5F and 5G show the results in the condition without a high cost (Fig 5E). As shown in these figures, the model successfully reproduces the experimental observations that DA depletion induced a preference reversal only in the condition with a high cost (Fig 5B and 5F) while increased the latency in both conditions (Fig 5C and 5G), although the subsequent recovery of the latency is not reproduced. Looking at the action values in the case with a high-cost (Fig 5D), the value of ‘Go’ to Arm 1 at the T-junction is fairly high before DA depletion. However, because this action is apart from reward, its value degrades quite prominently after DA depletion, becoming lower than the value of ‘Go’ to Arm 2, which is adjacent to reward (even though it is small reward). This explains the preference reversal (Fig 5B). In contrast, in the case without a high-cost (Fig 5H), the value of ‘Go’ to Arm 1 degrades only moderately after DA depletion, remaining higher than the value of ‘Go’ to Arm 2. In the meantime, in both conditions, initially there are value-contrasts between ‘Go’ and ‘Stay’ at States 1–3 but they degrade after DA depletion, explaining the increase in the latency (Fig 5C and 5G).

### The value-decay creates contrasts between ‘Go’ and ‘Stay’ values

**Fig 6:**
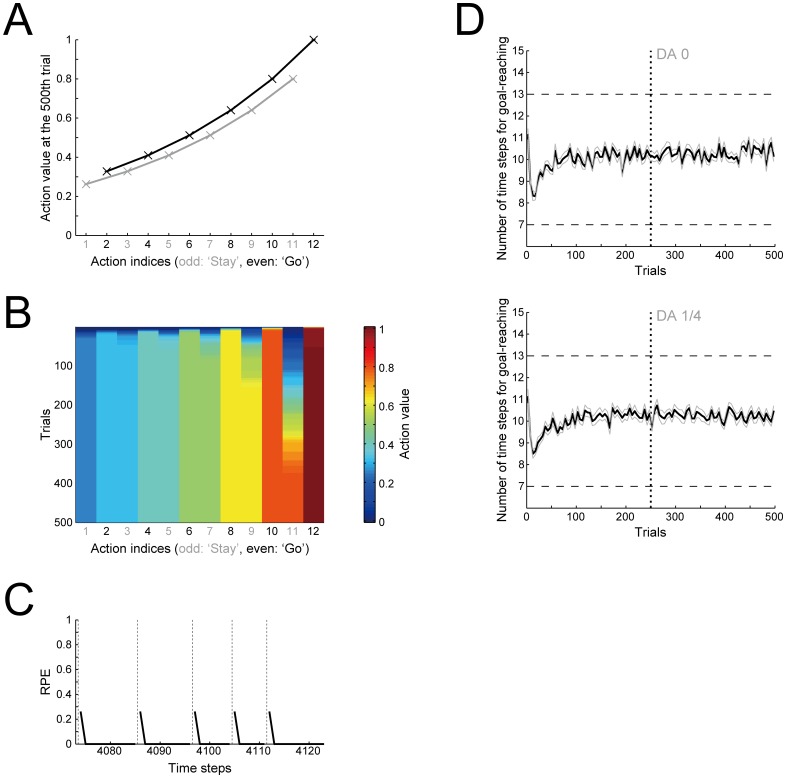
Simulation results without the value-decay but with temporal discounting of rewards (time discount factor *γ* = 0.8). **(A)** Action values at the end of the 500th trial. **(B)** Trial-by-trial changes of action values. **(C)** Examples of RPE. **(D)** Effects of post-training DA blockade on the number of time steps needed for goal-reaching. The configurations are the same as those in Fig 3A–3C or Fig 2C.

As we have shown above, the value-decay creates a gradient of ‘Go’ values towards the goal. It is known that temporal discounting of rewards also makes a gradient of values (c.f., [7]). However, we assumed no temporal discounting (i.e., time discount factor *γ* = 1) in the above simulations and thus the value-gradient observed in the above was caused solely by the value-decay. In order to compare the effects of the value-decay and the effects of temporal discounting, we conducted simulations of the original unbranched self-paced task (Fig 1) assuming no value-decay but instead temporal discounting (time discount factor *γ* = 0.8). Fig 6 shows the resulting action values (Fig 6A and 6B), RPE (Fig 6C), and the effect of DA blockade on the time needed for goal-reaching (Fig 6D). As shown in Fig 6A and 6B, a value-gradient is shaped, as expected. Contrary to the case with the value-decay, however, sustained positive RPE is generated only at the beginning of each trial (Fig 6C), and because of this, post-training blockade of DA signaling causes little effect on the subject speed (Fig 6D).

**Fig 7:**
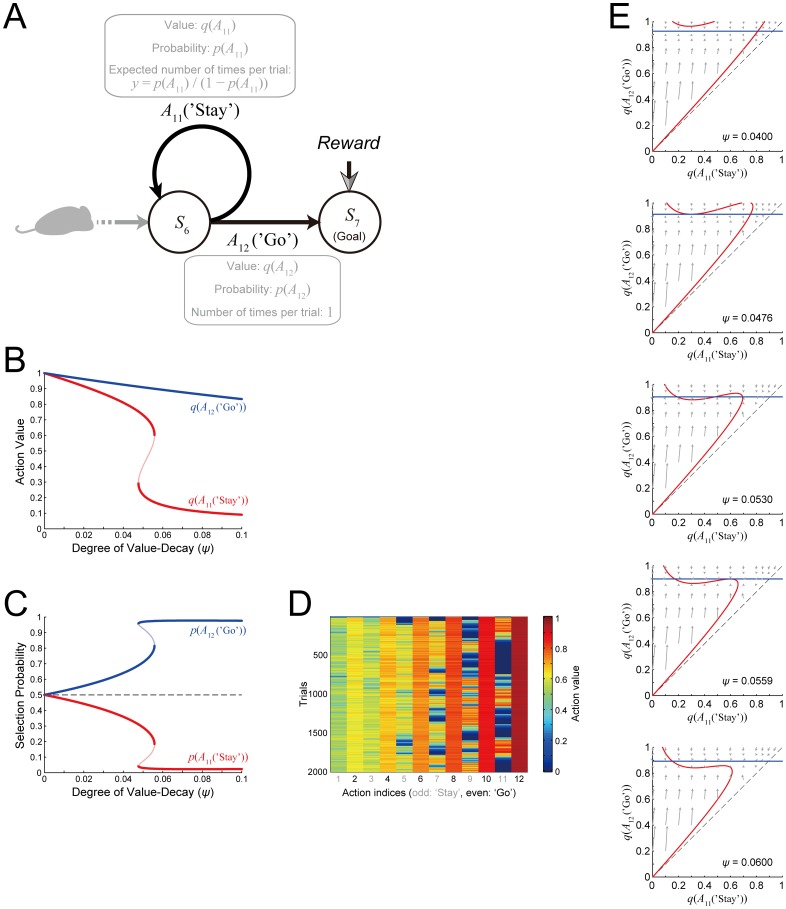
The value-decay generates value-contrasts between ‘Go’ and ‘Stay’. **(A)** Schematic diagram of the selection of *A*_11_ (‘Stay’) and *A*_12_ (‘Go’) at *S*_6_. We considered a reduced continuous-time dynamical system model that describes the time evolution of *q*(*A*_11_) and *q*(*A*_12_), which are continuous-time variables approximately representing the action values of *A*_11_ (‘Stay’) and *A*_12_ (‘Go’), respectively. **(B)** Bifurcation diagram of the reduced model, showing the equilibrium values of *q*(*A*_11_(‘Stay’)) (red line) and *q*(*A*_12_(‘Go’)) (blue line) (vertical axis) depending on the degree of the value-decay (horizontal axis; *ψ* = 0 corresponds to the case without the value-decay). Temporal discounting was not assumed. The thick parts of the lines indicate the stable equilibriums, whereas the thin part indicates the unstable equilibrium; the unstable equilibrium of *q*(*A*_12_(‘Go’)) is overlapped by the stable equilibrium and is thus invisible. **(C)** Probability of selecting *A*_11_ (‘Stay’) (red) or *A*_12_(‘Go’) (blue) at the equilibriums (vertical axis) depending on the degree of the value-decay (horizontal axis). The thick parts and thin parts correspond to the stable and unstable equilibriums, respectively. **(D)** A simulation result of the original model with the decay rate *φ =* 0.0045, in which there appears a phenomenon indicative of bistability: the value of *A*_11_ (‘Stay’) fluctuates between two levels in long time scales. **(E)** Phase diagrams in the cases with five different degrees of the value-decay. The red and blue lines indicate the nullclines on which the time derivative of *q*(*A*_11_(‘Stay’)) or *q*(*A*_12_(‘Go’)) is zero, respectively. The gray arrows indicate the direction of the time evolution of *q*(*A*_11_(‘Stay’)) and *q*(*A*_12_(‘Go’)) (indicating vectors (d*q*(*A*_11_)/d*t*, d*q*(*A*_12_)/d*t*)/2). Notably, the analysis of the reduced model was conducted under the assumption of *q*(*A*_11_(‘Stay’)) ≤ *q*(*A*_12_(‘Go’)), which corresponds to the upper left region of the black dashed line.

Comparing the value gradient caused by the value-decay (Fig 3A and 3B, middle/right) and the gradient caused by temporal discounting (Fig 6A and 6B), the differences of the action values between ‘Stay’ and ‘Go’ are much larger in the case with the value-decay. This is considered to be because, in the case with the value-decay, the values of unchosen actions just decay whereas those of chosen actions are kept updated according to RPE. In order to mathematically confirm this conjecture, especially, the long-term stability of such a large contrast between ‘Stay’ and ‘Go’ values, we considered a reduced dynamical system model of our original model, focusing on the last state preceding the goal (i.e., *S*_6_ in Fig 1), and conducted bifurcation analysis. Specifically, we derived a two-dimensional dynamical system that approximately describes the dynamics of the action values of *A*_11_ (‘Stay’) and *A*_12_ (‘Go’) at *S*_6_ (Fig 7A; see the Materials and Methods for details), and examined how the system's behavior qualitatively changes along with the change in the degree of the value-decay. Temporal discounting was not assumed (i.e., *γ* was assumed to be 1) in this reduced model so as to isolate the effect of the value-decay.

Fig 7B is the resulting bifurcation diagram showing the equilibrium action values of *A*_11_ (‘Stay’) and *A*_12_ (‘Go’) at *S*_6_ (with approximations) with the degree of the value-decay varied, and Fig 7C shows the probability of choosing *A*_11_ (‘Stay’) and *A*_12_ (‘Go’) at the equilibrium point. As shown in Fig 7B, it was revealed that as the degree of the value-decay increases, qualitative changes occur twice (in technical terms, arrangements of the nullclines shown in Fig 7E indicate that both of them are saddle-node bifurcations (c.f., [27])), and when the value-decay is larger than a critical degree (*ψ* ≈ 0.0559), there exists a unique stable equilibrium with a large contrast between the action values of *A*_11_ (‘Stay’) and *A*_12_ (‘Go’). It is therefore mathematically confirmed that the value-decay causes a large contrast between the steady-state action values of ‘Stay’ (*A*_11_) and ‘Go’ (*A*_12_) as conjectured in the above. Similar mechanism is considered to underlie the observed contrasts between ‘Stay’ and ‘Go’ values at the other states (Fig 3A and 3B, middle/right).

**Fig 8:**
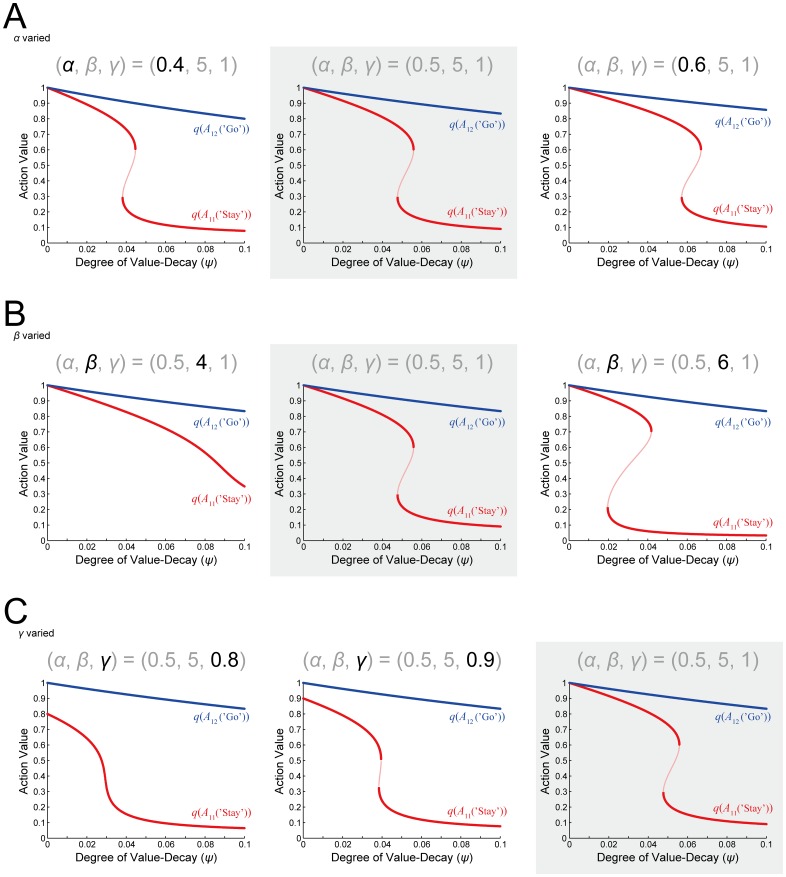
Dependence of the bifurcation diagram of the reduced model on the RL parameters. The three panels with the gray background are the same as Fig 7B (re-presented for comparison), showing the case with the standard RL parameter values: the learning rate *α* = 0.5, the inverse temperature *β* = 5, and the time discount factor *γ* = 1 (i.e., no temporal discounting). **(A)** The learning rate *α* was varied from the standard value 0.5 (middle panel). **(B)** The inverse temperature *β* was varied from the standard value 5 (middle panel). **(C)** The time discount factor *γ* was varied from the standard value 1 (right panel). The configurations are the same as those in Fig 7B.

Notably, the bifurcation diagram (Fig 7B) suggests that there exists bistability when the degree of the value-decay is within a certain range. We conducted a simulation of the original model with the decay rate *φ =* 0.0045, and found that there indeed appears a phenomenon indicative of bistability. Specifically, the value of ‘Stay’ (*A*_11_) was shown to fluctuate between two levels in long time scales (Fig 7D). Such bistability can potentially cause a hysteresis, in a way that learned values depend on the initial condition or the learning history, although the range of the degree of the value-decay for bistability is not large. Fig 8 shows the dependence of the bifurcation diagram on the RL parameters. As shown in the figure, the existence and the range of bistability critically depend on the inverse temperature (*β*) (representing the sharpness of soft-max selection) and the time discount factor (*γ*). The figure also indicates, however, that whether bistability exists or not, as the degree of the value-decay increases, there emerges a prominent contrast between ‘Stay’ and ‘Go’ values.

**Fig 9:**
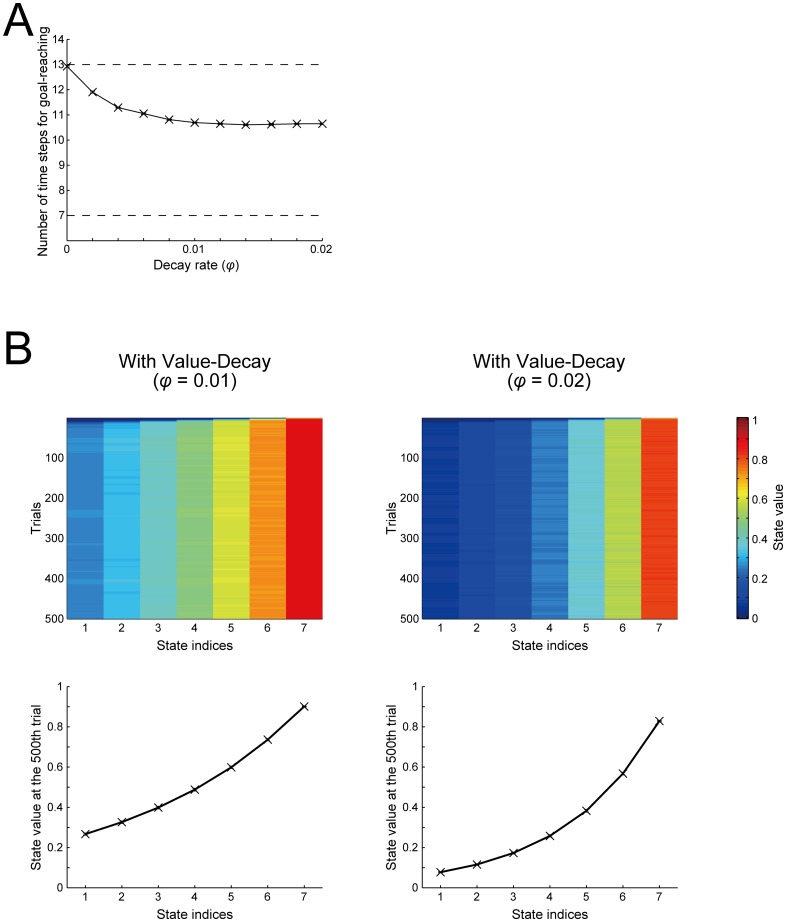
Effects of the value-decay in the cases in which action selection is based on the state values. **(A)** Number of time steps needed for goal-reaching averaged over 500 trials (vertical axis) in the cases with various decay rates (i.e., rates of decay of the state values) (horizontal axis). The configurations are the same as those in Fig 2A. **(B)** Trial-by-trial changes of the state values (top panels) and the state values at the end of the 500th trial (bottom panels) in the case with the decay rate *φ =* 0.01 (left) or 0.02 (right). The color indicates the state value averaged over 20 simulations, in reference to the rightmost color scale bar.

Importantly, it is considered that the facilitation of fast goal-reaching by the value-decay in the simulations shown so far is actually caused by the value-contrasts between ‘Stay’ and ‘Go’ rather than the gradient of ‘Go’ values explained before, because value-based choice is made between ‘Stay’ and ‘Go’ rather than between successive ‘Go’ actions. Nevertheless, the decay-induced value-gradient can indeed cause a facilitatory effect if selection of ‘Go’ or ‘Stay’ is based on the state values rather than the action values. Specifically, if our model is modified in the way that the probability of choosing ‘Go’ or ‘Stay’ depends on the value of the current and the next state (while action values are not defined: see the Materials and Methods for details), introduction of the decay of learned (state) values can still cause facilitation of goal-reaching (Fig 9A). Since the values of ‘Go’ and ‘Stay’ are not defined and thus the "value-contrast" appeared in the original model does not exist, this facilitation is considered to come from the gradient of state values (Fig 9B). Facilitation appears to be in similar levels as the decay rate changes from 0.01 to 0.02 (Fig 9A), and it is considered to be because, while the slope near the start becomes shallower, the slope near the goal becomes steeper (Fig 9B).

### Dependence of the effect of the value-decay on the RL parameters and algorithms

**Fig 10:**
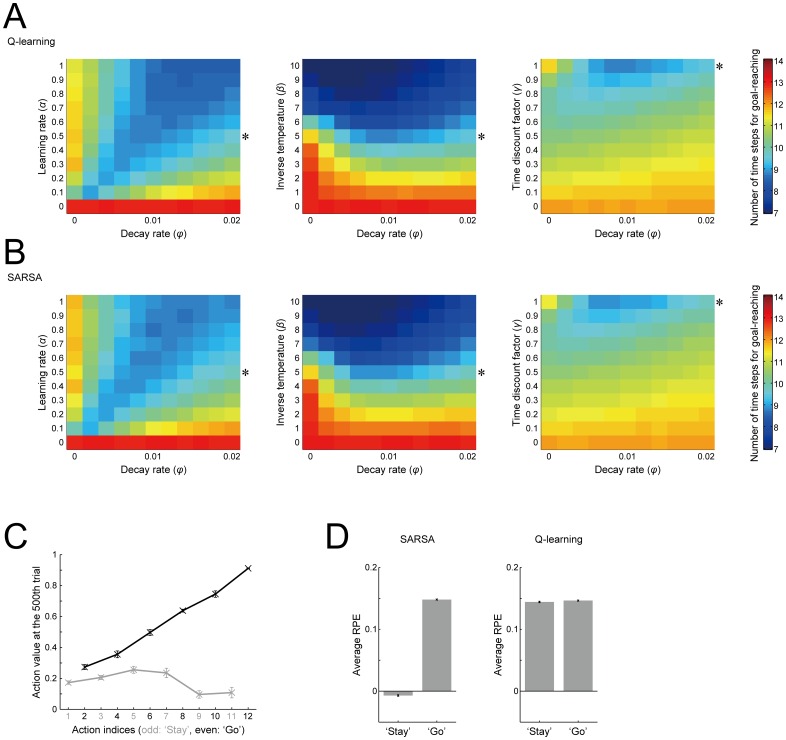
Dependence of the effect of the value-decay on the RL parameters and algorithms. **(A)** Dependence on the RL parameters. The color indicates the number of time steps needed for goal-reaching averaged over 500 trials, further averaged over 20 simulations, in reference to the rightmost color scale bar. The horizontal axis indicates the decay rate (*φ* = 0~0.02), and the vertical axis indicates the RL parameter that was varied: the learning rate *α* (left panel), inverse temperature *β* (middle panel), and time discount factor *γ* (right panel). The asterisks at the right edge of each panel indicate the standard RL parameter values used in the simulations shown in the previous figures unless otherwise described. **(B)** Results of the case where RPE was assumed to be calculated according to the SARSA algorithm rather than the Q-learning algorithm, which was assumed in the simulations/analyses shown so far (note that Q-learning-type RPE calculation was again assumed in the simulations/analyses in Figs 11–14). The configurations are the same as those in (A). **(C)** Action values of ‘Go’ (black lines/crosses) and ‘Stay’ (gray lines/crosses) at the end of the 500th trial in the case with SARSA-type RPE. The configurations are the same as those in Fig 3A. **(D)** Average RPE generated upon taking ‘Stay’ and ‘Go’ in the case assuming SARSA-type (left panel) and Q-learning-type (right panel) RPE.

We examined how the effect of the value-decay on fast goal-reaching depends on the RL parameters, specifically, the learning rate, the inverse temperature, and the time discount factor. Fig 10A shows the time needed for goal-reaching averaged over 500 trials in conditions varying one of the RL parameters and the decay rate. As shown in the figure panels, although a large inverse temperature (indicating an exploitative choice policy) realizes fast goal-reaching without the value-decay (middle panel of Fig 10A), facilitation of fast goal-reaching by introduction of the value-decay occurs within a wide range of RL parameters. Notably, the right panel of Fig 10A shows that the value-decay can realize faster goal-reaching than temporal discounting does, given that the other parameters are fixed to the values used here. This is considered to reflect that while both the value-decay and temporal discounting create a value-gradient from the start to the goal, only the value-decay additionally induces value-contrasts between ‘Stay’ and ‘Go’ as we have shown above.

In the results presented so far, we assumed in the model that RPE is calculated according to a major RL algorithm called Q-learning [28] (Eq (1) in the Materials and Methods), based on the empirical suggestions that DA neuronal activity in the rat ventral tegmental area (VTA) and DA concentration in the nucleus accumbens represent Q-learning-type RPE [21, 29]. However, there is in fact also an empirical suggestion that DA neuronal activity represents RPE calculated according to another major RL algorithm called SARSA [30] (Eq (2) in the Materials and Methods) rather than Q-learning in the monkey substantia nigra pars compacta (SNc) [31, 32]. It remains elusive whether such a difference comes from the differences in the species, regions, task paradigms or other conditions. We examined how the model's behavior changes if SARSA-type RPE is assumed instead of Q-learning type RPE. Fig 10B shows the time needed for goal-reaching averaged over 500 trials, with the RL parameters varied as before, and Fig 10C shows the learned values of each action at the end of 500 trials. As shown in the figures, it turned out that the effects of the value-decay, as well as the underlying value-gradient and value-contrast, are very similar to the cases with Q-learning type RPE.

There is, however, a prominent difference between the cases of SARSA and Q-learning. Specifically, in the case of SARSA, RPE generated upon taking ‘Go’ was much larger than RPE generated upon taking ‘Stay’ (Fig 10D, left), whereas there was no such difference in the case of Q-learning (Fig 10D, right). The difference in RPE between ‘Go’ and ‘Stay’ in the SARSA case is considered to reflect the value-contrast between the learned values of ‘Go’ and ‘Stay’ (Fig 10C). This is not the case with Q-learning because the Q-learning-type RPE calculation uses the value of the maximum-valued action candidates, which would be ‘Go’ in most cases, regardless of which action is actually selected. The SARSA-type RPE calculation, by contrast, uses the value of actually selected action (compare Eqs (1) and (2) in the Materials and Methods). The difference in RPE between ‘Go’ and ‘Stay’ in the SARSA case could potentially be related to a recent finding [33] that DA in the rat nucleus accumbens responded to a reward-predicting cue when movement was initiated but not when animal had to stay. However, our present model would be too simple to accurately represent the task used in that study and the neural circuits that are involved, and elaboration of the model is desired in the future.

### Reward-amount-dependences of the effect of the value-decay, subject's speed, and the average RPE

**Fig 11:**
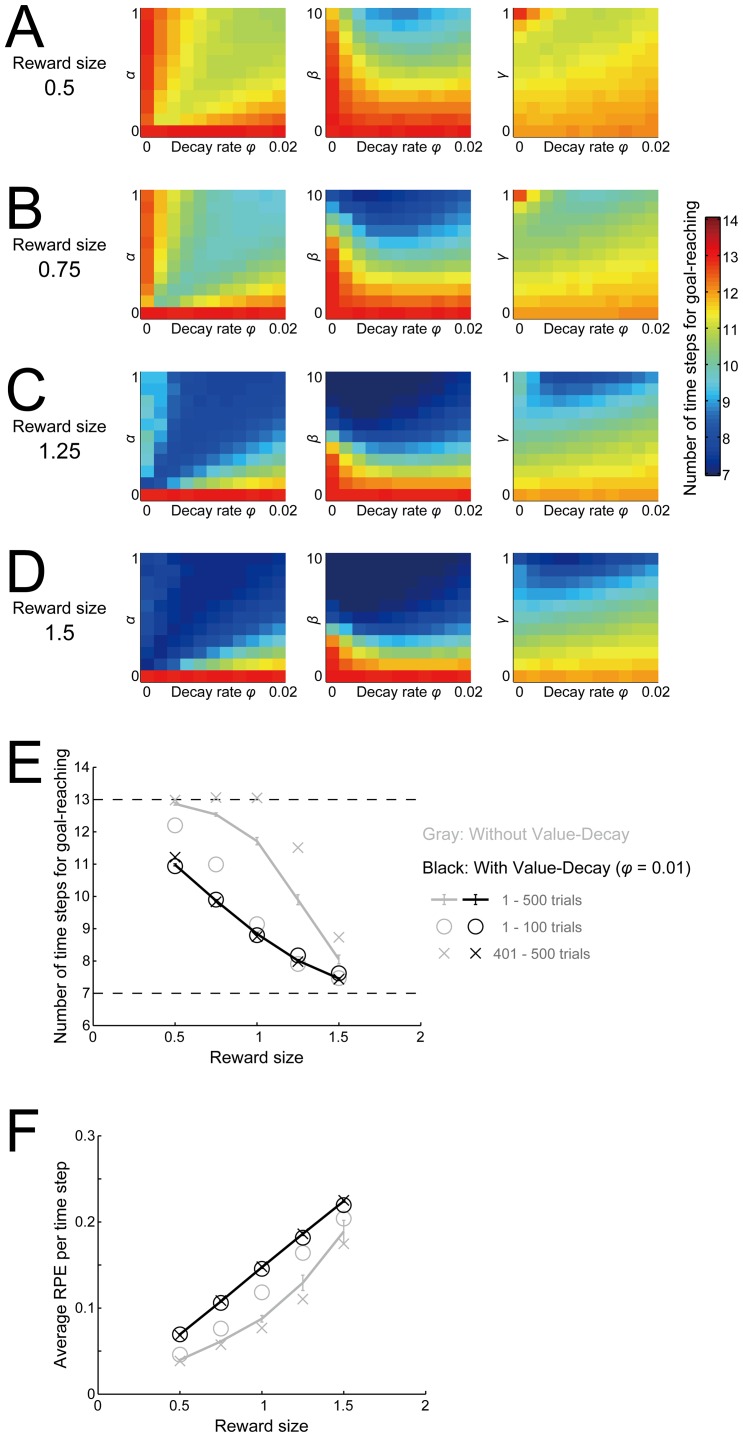
Reward-amount-dependences of the effect of the value-decay, subject's speed, and the average RPE. **(A-D)** The number of time steps needed for goal-reaching averaged over 500 trials for the cases with reward amount 0.5 (A), 0.75 (B), 1.25 (C), and 1.5 (D). The configurations are the same as those in Fig 10A. **(E,F)** Relationship between the reward amount and the average number of time steps for goal-reaching (E) or the average RPE per time-step (F), in the case with the standard values of RL parameters (i.e., *α* = 0.5, *β* = 5, and *γ* = 1) and the decay rate of *φ =* 0.01 (black symbols) or *φ =* 0 (gray symbols). The lines show the average over 500 trials, and the error bars indicate the mean ± SE of 20 simulations. The circles and the crosses show the average over 1–100 trials and 401–500 trials, respectively. The two dashed lines in (E) indicate the theoretical minimum (bottom) and the chance level (top).

We examined how the facilitatory effect of the value-decay depends on the amount of the reward obtained at the goal, which was fixed at *r* = 1 in the simulations so far presented (we again consider Q-learning-type RPE in the following). Fig 11A, 11B, 11C and 11D show the time needed for goal-reaching averaged over 500 trials, with the RL parameters varied as before, in the cases with reward amount 0.5, 0.75, 1.25, and 1.5, respectively. As shown in the figures, the overall tendency of the effect of the value-decay does not largely change across this threefold range of reward amount.

Meanwhile, the figures indicate that as the reward amount increases, the time needed for goal-reaching generally decreases, or in other words, the subject's speed increases. The black line in Fig 11E shows this relationship in the case with the standard RL parameters used so far and the decay rate of 0.01. As shown in this figure, there is a clear negative relationship between the reward amount and the time needed for goal-reaching. We also examined how the average RPE per time-step during 500 trials depends on the reward amount. As shown in the black line in Fig 11F, we found that there is a positive relationship between the reward amount and the average RPE. These negative and positive reward-amount-dependences of the time needed for goal-reaching and the average RPE, respectively, are in line with the experimental findings [7] that the subject's latency and the minute-by-minute DA level in the nucleus accumbens were negatively and positively related with the reward rate, respectively, given that RPE in our model is represented by DA as we assumed.

The commonality of the effect of the value-decay across the range of reward amount (Fig 11A–11D) and the positive reward-amount-dependence of the average RPE (Fig 11F, black line) are considered to appear because our model is largely scalable to (i.e., variables are scaled in proportion to) the changes in the reward amount except for the effect of the inverse temperature. The negative reward-amount-dependence of the time needed for goal-reaching (Fig 11E, black line) is considered to appear because as the reward amount increases, the overall magnitudes of learned values, and thereby also the value-contrasts between ‘Stay’ and ‘Go’, increase.

The gray lines in Fig 11E and 11F show the relationship between the reward amount and the time needed for goal-reaching (Fig 11E) or the RPE per time-step (Fig 11F) in the case without the value-decay, averaged over 500 trials. The gray circles and crosses in these figures show the averages for 1–100 trials and 401–500 trials, respectively. As shown in these, in the case without the value-decay, there are negative and positive reward-amount-dependences of the time needed for goal-reaching and the RPE per time-step in the initial phase, but such dependences gradually degrade along with trials. This is considered to be because the values of ‘Stay’ actions gradually increase toward the saturation (Fig 3B, left). In contrast, in the case with the value-decay (*φ =* 0.01), there are little differences in the time needed for goal-reaching and the RPE per time-step between 1–100 trials (black circles in Fig 11E and 11F) and 401–500 trials (black crosses in Fig 11E and 11F). This is reasonable given that gradual saturation of ‘Stay’ values does not occur in the case with the value-decay (Fig 3B, middle).

### Additional analyses (1): Dependence on the model architectures, and robustness to perturbations in reward environments

**Fig 12:**
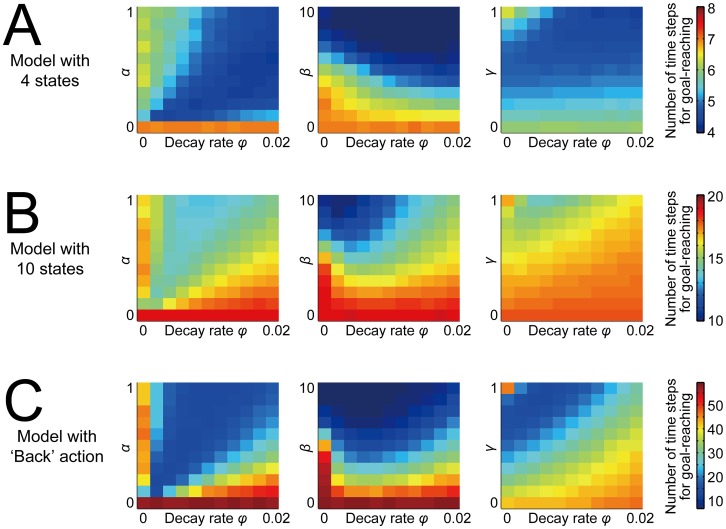
Dependence of the effect of the value-decay on the model architectures. **(A,B)** Results for the models with 4 (A) or 10 (B) states, including the start and the goal. **(C)** Results for the model (with 7 states) that incorporated ‘Back’ action, in addition to ‘Go’ and ‘Stay’, at each state except for the start and the goal. The configurations are the same as those in Fig 10A.

We further examined how the facilitatory effect of the value-decay depends on the architectures of the model, in particular, the number of states and the number of action candidates. Regarding the number of states, in the results so far shown, we assumed seven states, including the start and the goal, as shown in Fig 1. Fig 12A and 12B show the time needed for goal-reaching averaged over 500 trials in the cases with four or ten states, respectively. As shown in the figures, although the optimal decay rate that realizes fastest goal-reaching varies depending on the number of states, facilitation of fast goal-reaching by introduction of the value-decay can occur in either case.

Regarding the number of the action candidates, we have so far assumed that either of the two actions, ‘Go’ or ‘Stay’, can be taken at each state except for the goal (or the T-junction in the case of the T-maze). This can be a good model of certain types of self-paced tasks that are intrinsically unidirectional, such as pressing a lever for a fixed amount of times to get reward. However, there are also self-paced tasks that are more like bidirectional, for instance, movements in an elongated space with reward given at one of the ends. Such tasks might be better represented by adding ‘Back’ action to the action candidates at each state except for the start and the goal. Fig 12C shows the time needed for goal-reaching averaged over 500 trials in the case where the ‘Back’ action was added. As shown in this figure, while the time needed for goal-reaching is generally larger than the cases without the ‘Back’ action as naturally expected, the value-decay can facilitate fast goal-reaching in this case too.

**Fig 13:**
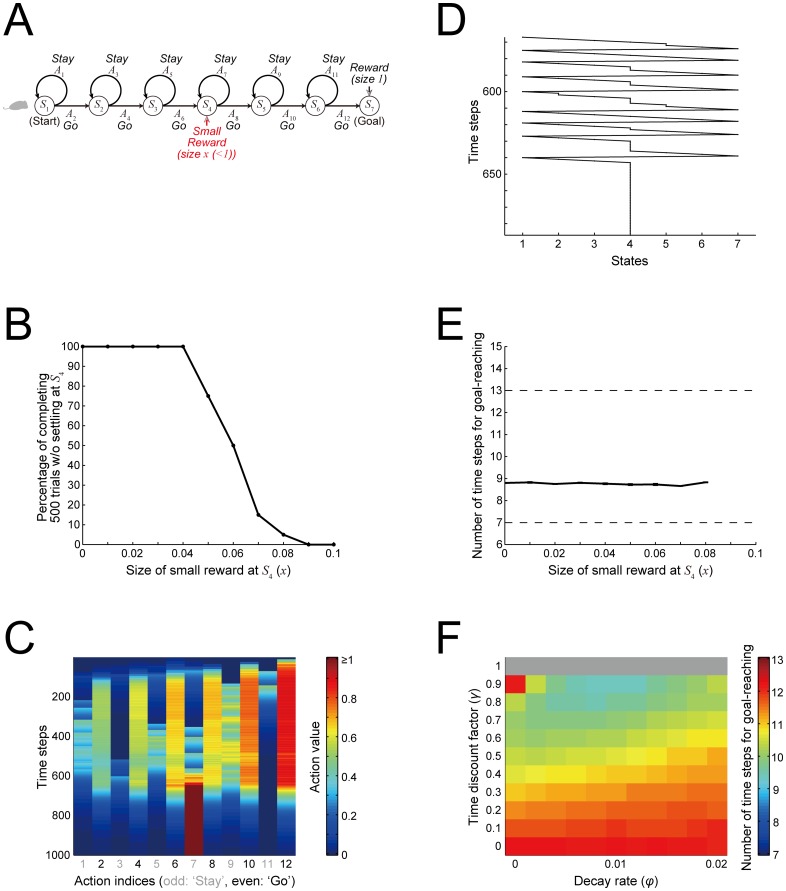
Robustness of the effect of the value-decay to perturbations in reward environments. **(A)** Simulated perturbation: small reward of size *x* (< 1, which is the amount of the reward given at the goal) is given at *S*_4_ whenever subject is located there (i.e., repeatedly at every time step if subject stays at *S*_4_). **(B)** Percentage of simulation runs (out of total 20 runs for each condition) in which subject completed 500 trials within 35000 time steps (i.e., within 70 time steps per trial on average) without settling at *S*_4_. **(C)** Time evolution of the action values in a simulation run with *x* = 0.1 in which subject settled at *S*_4_ before completing 500 trials. The color indicates the action value in reference to the rightmost color scale bar: note that the color is saturated for values ≥ 1. The vertical axis indicates the time steps (from top to bottom) and the horizontal axis indicates the indices of the actions (odd/gray: ‘Stay’, even/black: ‘Go’: Fig 13A). At around time-step 650, the value of *A*_7_ (i.e., ‘Stay’ at *S*_4_) became very large while the values of the other actions decayed out, indicating that subject settled at *S*_4_. **(D)** The subject's state transitions in the simulation run shown in (C) around time-step 650, showing that the subject indeed settled at *S*_4_ around this time. **(E)** Number of time steps needed for goal-reaching averaged over 500 trials. The solid line with error bars indicates the mean ± SE for the simulation runs in which 500 trials were completed. The two dashed lines indicate the theoretical minimum (bottom) and the chance level (top). **(F)** Simulation results with *x* = 0.1 for the cases with both the value-decay (horizontal axis) and temporal discounting (vertical axis). The color indicates the number of time steps needed for goal-reaching averaged over 500 trials, further averaged over 20 simulations, in reference to the rightmost color scale bar: the gray zone at the top (*γ* = 1) indicates that, in these conditions (i.e., without temporal discounting), subject did not complete 500 trials.

It is also a question of how robust the effect of the value-decay is to perturbations in reward environments. In particular, given that the values of unchosen actions just decay, it is conceivable that, if small reward is given at a state between the start and the goal (e.g., *S*_4_: Fig 13A) whenever subject is located there (i.e., repeatedly at every time step if subject stays at *S*_4_), subject might learn to stay there persistently rather than to reach the goal. Denoting the size of the small reward by *x* (< 1, which is the amount of the reward given at the goal), if 7*x* < *x* + 1 ⇔ *x* < 0.166…, such a persistent stay is however inferior to the fastest repetition of goal-reaching in terms of the average reward obtained per time-step. We examined the behavior of modeled subject when small reward is given at *S*_4_ with its size *x* varied from 0 to 0.1, in the case with the value-decay (*φ =* 0.01). Fig 13B shows the resulting percentage of simulation runs (out of total 20 runs for each condition) in which subject completed 500 trials within 35000 time steps (i.e., within 70 time steps per trial on average) without settling at *S*_4_. As shown in the figure, the percentage for the completion of 500 trials is 100% when the size of the reward at *S*_4_ is ≤ 0.04, whereas the percentage then decreases as the size of the reward at *S*_4_ further increases. This indicates that a persistent stay at *S*_4_ actually occurs even if it is not advantageous: Fig 13C and 13D show such an example. Fig 13E shows the number of time steps needed for goal-reaching averaged over 500 trials, only for the simulation runs completing 500 trials in the cases where the completion rate is less than 100%. As shown in the figure, the speed of goal-reaching is kept fast, comparable to the case without reward at *S*_4_ (i.e., *x* = 0). These results indicate that the facilitatory effect of the value-decay on fast goal-reaching has a certain degree of tolerance to this kind of perturbation in reward environments, although it eventually fails as the perturbation becomes larger.

Nonetheless, when temporal discounting (*γ* = 0.9, 0.8, …) was also assumed in the model with the small reward *x* = 0.1 at *S*_4_, persistent stay at *S*_4_ before completing 500 trials was not observed in 20 simulation runs for each of the tested decay rates, and the value-decay could have facilitatory effects (Fig 13F). The absence of persistent stay at *S*_4_ is considered to be because the value of ‘Stay’ at *S*_4_ is bounded due to temporal discounting. For example, in the case with *γ* = 0.9 and no value-decay, if the subject keeps staying at *S*_4_, the value of ‘Stay’ at *S*_4_ converges to 1 (solution of the equation of *V*: 0 = 0.1 + 0.9*V* − *V*). This is still larger than the convergence value of ‘Go’ at *S*_4_, which is 0.9^2^ = 0.81. However, since the growth of the ‘Stay’ value from the initial value 0 is likely to be slower than the growth of the ‘Go’ value, subject would rarely begin to settle at *S*_4_. In contrast, in the case with no temporal discounting and no value-decay, if the subject keeps staying at *S*_4_, the value of ‘Stay’ at *S*_4_ increases unboundedly, leading to a persistent stay. Actually, the value-decay also bounds the value of ‘Stay’ at *S*_4_, but its effect is weak when the decay rate is small as we have so far assumed. For example, in the case with no temporal discounting, *φ =* 0.01, and the learning rate *α* = 0.5, if the subject keeps staying at *S*_4_, the value of ‘Stay’ at *S*_4_ converges to 4.95 (solution of the equation of *V*: *V* = (1 − 0.01)(*V* + 0.5×0.1)), which is fairly large. In this way, temporal discounting effectively prevents the subject from settling at *S*_4_. The value-decay can then facilitate fast goal-reaching by creating the value-contrast between ‘Go’ and ‘Stay’.

### Additional analyses (2): Elaboration of the model towards accurate reproduction of behavioral profiles

So far we have assumed that subject exists in one of the discrete set of states, and selects either ‘Go’ or ‘Stay’, moving to the next state or staying at the same state. Given this simple structure, our model can potentially represent a variety of self-paced behavior, from spatial movement to more abstract Go/No-Go decision sequences. At the same time, however, our model is likely to be too simple to accurately model any specific behavior. In particular, in the case of spatial movement, subject does not really exist only in one of a small number of locations, and would not abruptly stop or literally ‘stay’ at a particular location. Meanwhile, subject should stop or slow down in the face of a physical constraint (e.g., the start, the junction, or the end of a maze) or a salient event (e.g., reward) as observed in experiments [6]. An emerging question is whether our model can be extended to reproduce these observations while preserving its main features.

**Fig 14:**
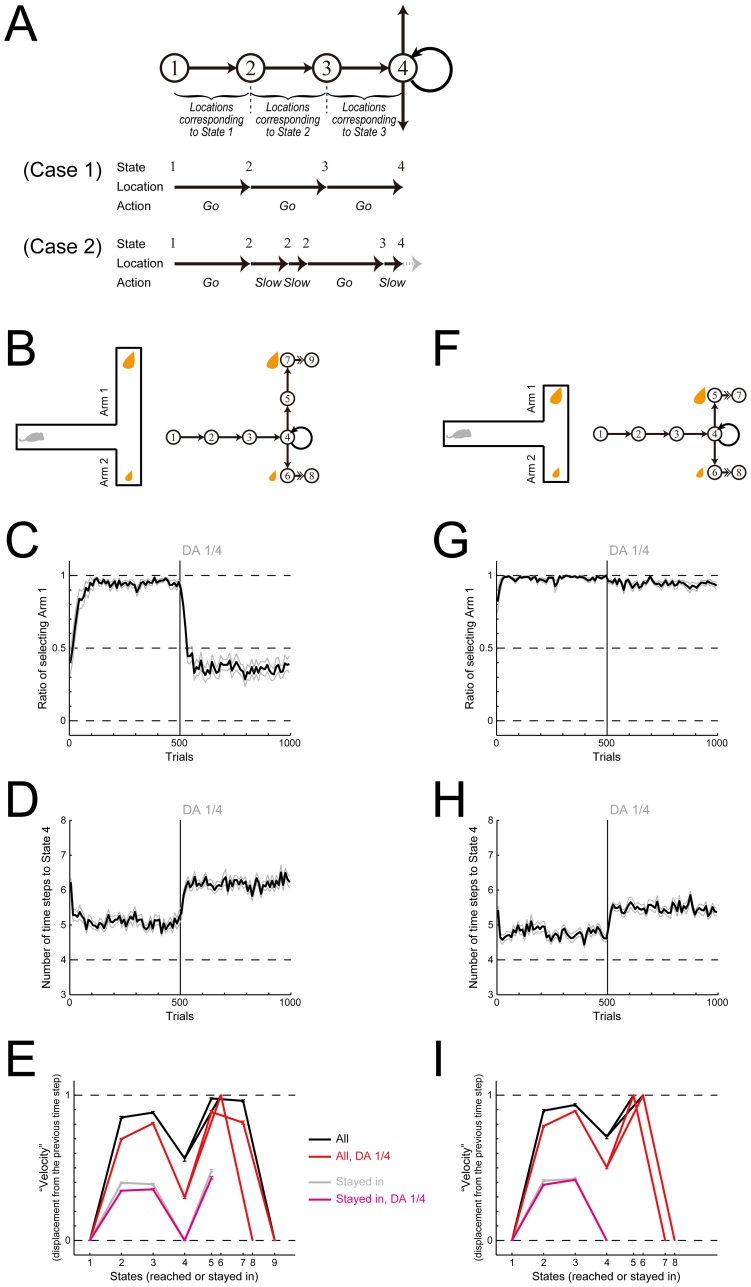
Simulations of the cost-benefit decision making task in a T-maze by an elaborated model, aiming at reproducing the velocity profiles observed in a (different) T-maze task. **(A)** Schematic explanation of the elaborated model. The one-to-one correspondence between the subject's physical location and the internal state assumed in the original model was changed into a loose coupling, in which each state corresponds to a range of physical locations as illustrated. At each time step, subject at a given location chooses either ‘Go’ or ‘Slow’, except that the subject is at the start, the T-junction, or the reward location (in the ends of the T-maze). By selecting ‘Go’, subject moves straightforward for a time step with the "velocity" 1, meaning that the subject's physical location is displaced by 1, unless there is a physical constraint. By selecting ‘Slow’, subject moves straightforward for a time step with the "velocity" halved, meaning that the subject's physical location is displaced by the half of the displacement during the previous time interval, unless there is a physical constraint. At the start (State 1), subject was assumed to take ‘Go’ or ‘Stay’, and at the T-junction (State 4), subject was assumed to take ‘Go’ to either of the two arms or ‘Stay’, in the same manners as in the original model. (Case 1) shows the case where subject at the start point chooses ‘Go’ three times in succession, whereas (Case 2) shows the case where subject chooses ‘Go’, ‘Slow’, ‘Slow’, ‘Go’, and ‘Slow’. Notably, in Case 2, subject transitions from State 3 to State 4 by choosing ‘Slow’ rather than ‘Go’. **(B)** Schematic diagram of the task condition where there are high-cost-high-return and low-cost-low-return options. When reaching reward, subject is assumed to take the consummatory action (indicated by double-lined arrows). **(C,D)** Ratio of choosing the large-reward arm (Arm 1) (C) and the average number of time-steps towards the T-junction (State 4) (D). DA depletion (to the quarter of the original) after 500 trials was simulated as before. The configurations are the same as those in Fig 5B and 5C. **(E)** Average "velocity", i.e., displacement from the previous time step, when subject reached or stayed in each state (horizontal axis). The black and red solid lines indicate the "velocity" averaged across all the cases in 251–500 trials (before DA depletion) and 751–1000 trials (after DA depletion), respectively. The gray and magenta lines indicate the "velocity" averaged across the cases where subject stayed in the state at the previous and current time steps: notably, because of the decoupling of the physical location and the internal state, subject can still move. The error bars indicate the mean ± SE of 20 simulations (black and red) or of simulations (out of the total 20) which had the corresponding data (gray and magenta). **(F-I)** Same as (B-E) for the different task condition where there are low-cost-high-return and low-cost-low-return options.

In order to examine this, we developed an elaborated model of self-paced spatial movement in the T-maze. In this model, the exact one-to-one correspondence between the subject's physical location and the internal state assumed in the original model was changed into a loose coupling, in which each state corresponds to a range of physical locations (Fig 14A). Also, ‘Stay’ action in the original model was replaced with ‘Slow’ action unless there is a physical constraint (i.e., the start, the T-junction, or the end). By selecting ‘Slow’, subject moves straightforward for a time step with the "velocity" halved from the previous time step (or further decreased when there is a physical constraint). ‘Slow’ was introduced to eliminate the abrupt/complete stop appeared in the original model, and mechanistically, it can represent inertia in decision and/or motor processes [34, 35]. With these modifications, state transitions can sometimes occur even when subject chooses ‘Slow’ rather than ‘Go’ (Fig 14A, Case 2), different from the original model. At the T-junction, subject was assumed to take ‘Go’ to either of the two arms or ‘Stay’ in the same manner as in the original model. At the reward location, subject was assumed to take the consummatory action for a time step (indicated by the double-lined arrows in Fig 14B and 14F), and proceed to the end state.

Using this elaborated model (see the Materials and Methods for details), we simulated the T-maze cost-benefit decision making task with DA depletion [24] that was simulated by the original model before (Fig 5). Fig 14C and 14D show the simulation results about the ratio of choosing the large-reward arm (Arm 1) and the average time needed for reaching the T-junction in the task conditions with high cost in the large-reward arm (Fig 14B), respectively. Fig 14G and H show the results in the task conditions without high cost in the large-reward arm (Fig 14F). As shown in the figures, the experimentally observed effects of DA depletion, i.e., the severe impairment of high-cost-high-return choice but not low-cost-high-return choice (Fig 14C and 14G) and the slowdown in both conditions (Fig 14D and 14H), can be reproduced by the elaborated model, as well as by the original model (Fig 5). Simultaneously, the elaborated model can also reproduce the velocity profiles observed in a (different) T-maze task [6], specifically, the slowdown and stop at the T-junction and the end of the maze and the absence of complete stop in the other locations (Fig 14E and 14I). This exemplifies the potential of our original model to be extended to accurately represent specific self-paced behavior.

## Discussion

We have shown that the value-decay in RL can realize sustained fast goal-reaching in a situation requiring self-paced approach towards a goal, modeled as a series of ‘Go’ or ‘No-Go’ (or ‘Stay’) selections. The underlying potential mechanisms turned out to be twofold: (1) a value-gradient towards the goal is shaped by value-decay-induced sustained positive RPE, and (2) value-contrasts between ‘Go’ and ‘Stay’ are generated because chosen values are continually updated whereas unchosen values simply decay. We have then shown that our model with the value-decay can provide potential mechanistic explanations for the key experimental findings that suggest the DA's roles in motivation, under the parsimonious assumption that the representation of RPE is the sole reward-related role of DA. Specifically, our model explains the (i) slowdown of self-paced behavior by post-training blockade of DA signaling [14] (Fig 2C), (ii) severe impairment of effortful actions to obtain rewards, but not of seeking of easily obtainable rewards, by DA blockade [11, 24] (Figs 5 and 14), and (iii) relationships between the reward amount, the level of motivation reflected in the speed of behavior, and the average level of DA [7] (Fig 11E and 11F). Simultaneously, our model also explains the various temporal patterns of DA signals (Fig 3C), confirming and extending the suggestion previously made by the non-self-paced model [23]. Moreover, the simulation results of the SARSA-version of our model could also potentially account for the recent finding [33] that DA ramping occurred when movement was initiated but not when animal had to stay (Fig 10D).

### Dopamine, RPE, and motivation

The notion that DA represents RPE has been supported by electrophysiological [1, 4], FSCV [2, 3, 36] and neuroimaging [37–39] results. Recently, optogenetic manipulations of DA neurons causally demonstrated the DA's role in representing RPE [40, 41]. On the other hand, pharmacological blockade of DA signaling has been shown to cause motivational impairments such as slowdown of behavior [14]. Crucially, such effects have been observed even when DA signaling was blocked after animals were well trained and RPE-based learning had presumably already been completed. These motivational effects have thus been difficult to explain by the notion that DA represents RPE, unless different function of DA was also assumed [42, 43].

Given such situations, Niv and colleagues [15] proposed a hypothesis that while DA's phasic response encodes RPE, DA's tonic concentration represents the average reward rate per unit time. They argue that as the reward rate decreases, optimal action speed should also decrease because the opportunity cost for not acting becomes relatively smaller than the extra cost for quickly acting, explaining why DA blockade causes slowdown. Extending this hypothesis, Lloyd and Dayan [16] proposed that quasi-tonic DA represents the expected amount of time discount of the value of next state caused by postponing action to get to the next state. This can explain the experimentally observed ramping DA signals [5–8] as reflecting a gradient of state values created by temporal discounting (as in our Fig 6A and 6B), also consistent with the arguments by [7]. These normative hypotheses, at the Marr's levels of computation and algorithm [44, 45], provide intriguing predictions that are desired to be experimentally tested. Meanwhile, it is also important to explore the Marr's level of implementation, namely, circuit/synaptic operations, which could potentially provide inspirations for the upper levels and *vice versa* [45]. The abovementioned normative hypotheses highlight essential issues at the circuit/synaptic level, including how the sustained DA signals are generated in the upstream and utilized in the downstream, how the selection of action timing is implemented, and how temporal discounting is implemented.

In our model, sustained DA signals are assumed to represent RPE, and thus the upstream and downstream mechanisms of sustained DA signaling should be nothing more than the mechanisms of how RPE is calculated in the upstream of DA neurons and how RPE-dependent value-update occurs through DA-dependent synaptic plasticity. Both of these mechanisms for RPE have been extensively explored (e.g., [46, 47]) and have now become clarified [17–20]. Regarding the selection of action timing, we assumed that it consists of a series of selections of two actions, ‘Go’ and ‘Stay’. We could thus assume general mechanisms of action selection, for which implementation has been explored [48–52] with empirical supports [50, 53, 54], although this leaves an important issue regarding how time is represented. As for the implementation of temporal discounting, we will discuss it below, in relation to the value-decay that can be implemented as decay of the plastic changes of the synaptic strengths.

There exists a different model that has also tried to give a bottom-up unified explanation of both the learning and motivation roles of DA, referring to circuit architectures of the basal ganglia [55]. However, although this model captures a wide range of phenomena, there are several potential issues or limitations. Firstly, this model assumes that phasic DA represents a simple form of RPE, called the Rescorla-Wagner prediction error [56], which lacks the upcoming-value term. However, RL models of the DA system, including our present model, widely assume the more complex form of RPE called the temporal difference (TD) RPE or TD error [25] (see [57] for detailed explanation) because there is a wealth of empirical supports that DA signals represent TD-RPE [1, 20, 58]. Secondly, because this model assumes the Rescorla-Wagner, rather than TD-, RPE, this model cannot describe the learning of the values of a series of actions or states, nor the changes of RPE, within a trial. As a corollary to this, this model does not explain the experimentally observed sustained DA signals [5–8, 21, 22]. Lastly, this model assumes that the two major basal ganglia pathways, the direct and indirect pathways, are associated with positive and negative reinforcement, respectively. Although this assumption is based on several lines of empirical results, alternative possibilities [43, 46, 47, 59, 60] have also been proposed for the operations of these pathways.

### Decay/forgetting of learned values in reinforcement learning

Decay, or forgetting, is apparently wasteful. However, recent work [61] has suggested that decay/forgetting is in fact necessary to maximize future rewards in dynamic environments. Even in a static environment, potential benefit of decay/forgetting has been pointed out [62]. There is also a study [63] that considered decay to explain features of extinction. Forgetting for capturing extinction effects was also assumed in the model that we have discussed right above [55]. However, the authors clearly mentioned that they "assumed some forgetting" "to capture overall extinction effects" and "none of the results are qualitatively dependent on" the parameter for forgetting. Therefore, their work should not have anything to do with the effects of forgetting explored in our present work. Along with these theoretical/modeling works, it has been suggested that RL models with decay could fit the experimental data of human [64–66], monkey [67], and rat [68] choice behavior potentially better than models without decay. Moreover, existence and benefits of decay/forgetting have also been suggested in other types of learning [69, 70].

Nonetheless, decay of learned values (value-decay) is not usually considered in RL model-based accounts of the functions of DA and cortico-basal ganglia circuits. RL models typically have the time discount factor and the inverse temperature (representing choice sharpness) as major parameters [25]. Temporal discounting generates a value-gradient (Fig 6A and 6B) [7, 16], and is suggested [71] to ensure that maximizing rewards simultaneously minimizes deviations from physiologically desirable states. Gradually increasing the inverse temperature, i.e., choice sharpness, is known to be good for global optimization [72]. Possible neural implementation of these parameters have been explored [46, 73–75]. However, it is not sure whether these parameters are actually biologically implemented in their original forms. We have shown that the value-decay can generate a value-gradient, and also value-contrasts which lead to a sharp choice of ‘Go’. Choice-sharpening effect of decay is implied also in previous studies [62, 66]. These indicate a possibility that the value-decay, or its presumed biological substrate, synaptic decay, might in effect partially implement the parameters for temporal discounting and inverse temperature. In this sense, the suggestions that sustained DA represents/reflects time-discounted state values [7, 16] and our value-decay-based account are not necessarily mutually exclusive. Apart from temporal discounting and the inverse temperature, there is an additional note. There have been suggestions [34, 35] that animal's and human's decision making can be affected by the subject's own choice history, which is not included in standard RL models. The value-decay assumed in our model is expected to cause a dependency of decision making on choice history. Whether it can (partly) explain experimentally observed choice patterns would be an interesting issue to explore.

### Limitations and testable predictions

If the rate of the value-decay is always constant, after subject interrupts performing the task for a long period, learned values eventually diminish almost completely. Therefore, in order for our model to be valid, some sort of context-dependence of the value-decay needs to be assumed. There are several empirical implications. At the synaptic level, conditional synaptic decay depending on NMDA receptor-channels [76] or DA (in drosophila) [77] has been found. Behaviorally, memory decay was found to be highly context-dependent in motor learning [78]. More generally, it is widely observed that reactivation of consolidated memories makes them transiently labile [79]. With these in mind, we assume that the value-decay occurs when and only when subject is actively engaged in the relevant task/behavior. However, this issue awaits future verification.

There is also an important limitation of our present model regarding the explanatory power for the experimental observations. Specifically, as mentioned before, our model explains the increase in the latency caused by DA depletion in the cost-benefit decision making task in a T-maze [24], but does not explain the subsequent recovery of the latency. This recovery could possibly be explained if some slow compensatory mechanisms are additionally assumed in the model. It is important in future work to elaborate the model to account for this issue, as well as a diverse array of experimental observations on the DA's roles in motivation that are not dealt with in the present work.

There are also many open issues in the model, both the functional ones and the structural ones. The functional issues include how the states and the time are represented [80, 81] and how ‘Go’ and ‘Stay’ (or ‘No-Go’ or ‘Slow’) are represented. As for the latter, while ‘Go’ and ‘Stay’ might be represented as two distinct actions, ‘Stay’ could instead be represented as disengagement of working-memory/attention as proposed in a recent work [82]. The structural issues include, among others, how different parts of the cortico-basal ganglia circuits and different subpopulations of DA neurons cooperate or divide labor [83–90]. Regarding this, a recent study [91] has shown that DA axons conveying motor signals are largely different from those conveying reward signals and that the motor and reward signals are dominant in the dorsal and ventral striatum, respectively. DA in our model is assumed to represent RPE, and it should thus be released from the axons conveying reward signals that are dense in the ventral striatum. Even with this specification, the structure of our model is still quite simple, and exploring whether and to what extent the present results can be extended to models with rich dynamics at the levels of circuits (in the cortex [48, 50, 92–96], the striatum [97–103], the DAergic nuclei [104], and the entire cortico-basal ganglia system [49, 51, 105–114]), neurons [115, 116], and synapses [117–120] would be important future work.

Our model provides predictions that can be tested by various methods. First, if sustained DA signals indeed represent value-decay-induced sustained RPE, rather than being caused by other reasons [16, 121], the rate of the value-decay estimated from fitting of measured DA signals by our model should match the decay-rate estimated behaviorally. Behavioral estimation of decay-rate would be possible by preparing two choice options that are initially indifferent, manipulating the frequencies of their presentations, and then examining whether, and to what degree, less-frequently-presented option will be chosen less frequently. On the other hand, if sustained DA signals represent time-discounted state values [7, 16], time discount factor estimated from model-fitting of measured DA signals is expected to match behavioral estimation, e.g., from intertemporal choices. Note, however, that the value-decay and temporal discounting might not be completely distinct entities; the value-decay could be a partial implementation of temporal discounting (and the inverse temperature) as we discussed before.

Second, our model predicts that the strengths of cortico-striatal synapses are subject to decay in a context-dependent manner. This could be tested by measuring structural plasticity [18] during learning tasks (across several sessions and intervals). Our model further predicts that manipulations of synaptic decay affect DA dynamics and behavior in specific ways. It has been indicated that a protein kinase that is constitutively active, protein kinase M*ζ* (PKM*ζ*), is necessary for maintaining various kinds of memories, including drug reward memory in the nucleus accumbens [122]. Specifically, inhibition of PKM*ζ* in the nucleus accumbens core by injecting a selective peptide inhibitor has been shown to impair long-term drug reward memory [122]. It has also been shown that overexpression of PKM*ζ* in the neocortex enhances long-term memory [123]. We predict that overexpression of PKM*ζ* in the nucleus accumbens (ventral striatum) enhances reward memory, or in other words, reduces the value-decay, and thereby diminishes sustained DA signals and impairs goal-approach through the mechanisms described in the present work. Apart from PKM*ζ*, it has also been indicated that DA is required for transforming the early phase of long-term potentiation (LTP), which generally declines, into the late phase of LTP in the hippocampus [124, 125]. Similar DAergic regulation of the stability of LTP could potentially exist in the striatum that is the target of the present work, and if so, the decay rate could be manipulated by DA receptor agonists or antagonists. In the striatal synapses, however, DA signaling would be required for the induction of potentiation before its maintenance, as we have actually assumed in our model. Therefore, it would be necessary to explore ways to specifically manipulate maintenance (decay rate) of potentiation.

### Concluding remarks

The results of the present study suggest that when biological systems for value-learning are active (i.e., when subject is actively engaged in the relevant task/behavior) even though learning has apparently converged, the systems might be in a state of dynamic, rather than static, equilibrium where decay and update are balanced. As we have shown, such dynamic operation can potentially facilitate self-paced goal-reaching behavior, and this effect could be seen as a simple biologically plausible, though partial, implementation of temporal discounting and simulated annealing. It is also tempting to speculate that value-decay-induced sustained RPE might be subjectively felt as sustained motivation, considering recently suggested relationship between RPE and subjective happiness [126, 127]. This is in accordance with the suggestion that DA signals subjective reward value [128, 129], or more precisely, "utility prediction error" [130]. Despite that dynamic operation has these potential advantages, however, there can also be disadvantages. Specifically, continual decay and update of values must be costly, especially given that DA signaling is highly energy-consuming [131]. This could potentially be related to neuropsychiatric and neurological disorders, in particular, Parkinson's disease [131, 132], which is characterized by motor and motivational impairments that are suggested to be independently associated with DA [133]. Better understanding of the dynamic nature of biological value-learning systems will hopefully contribute to clinical strategies against these diseases.

## Materials and Methods

### Modeling self-paced operant task by reinforcement learning with value-decay/forgetting

We posited that behavioral task requiring self-paced voluntary approach (whether spatially or not) towards a goal can be represented as a series of ‘Go’ or ‘Stay’ (‘No-Go’) selections as illustrated in Fig 1. Discrete states (*S*_1_ ~ *S*_7_) and time steps were assumed. In each trial, subject starts from *S*_1_. At each time step, subject can take one of two actions, specifically, ‘Go’: moving to the next state or ‘Stay’: staying at the same state. Subject was assumed to learn the value of each action (‘Go’ or ‘Stay’) by a temporal-difference (TD) reinforcement learning (RL) algorithm incorporating the decay of learned values (referred to as the ‘value-decay’ below) [23], and select an action based on their learned values in a soft-max manner [134].

Specifically, at each time step (*t*), TD reward prediction error (RPE) *δ*(*t*) was assumed to be calculated according to the algorithm called Q-learning [28], which has been suggested to be implemented in the cortico-basal ganglia circuit [21, 43, 59], as follows:

$$\delta \left(t\right)=R\left(S\left(t\right)\right)+\gamma {\text{max}}_{A_{cand}\left(t\right)}\left\{Q\left(A_{cand}\left(t\right)\right)\right\}-Q\left(A\left(t-1\right)\right),$$ (1)

where *S*(*t*) represents the state where subject exists at time step *t*. *R*(*S*(*t*)) represents reward obtained at *S*(*t*), which is *r* (> 0) when *S*(*t*) = *S*_7_ (goal) and 0 at the other states, unless otherwise described. "*Q*(*A*)" generally represents the learned value of action *A*. *A*_*cand*_(*t*) represents the candidate of action that can be taken at time step *t*: when *S*(*t*) = *S*_*i*_ (*i* = 1, 2, …, 6), *A*_*cand*_(*t*) = *A*_2*i*−1_(‘Stay’) or *A*_2*i*_(‘Go’); when *S*(*t*) = *S*_7_ (goal), candidate of action was not defined and the term $\gamma {\text{max}}_{A_{cand}\left(t\right)}\left\{Q\left(A_{cand}\left(t\right)\right)\right\}$ was replaced with 0. *A*(*t* − 1) represents the action taken at time step *t* − 1; at the beginning of each trial, *A*(*t* − 1) was not defined and the term *Q*(*A*(*t* − 1)) was replaced with 0 so as to represent that the beginning of trial is not predictable. *γ* is the time discount factor (0 ≤ *γ* ≤ 1). In a separate set of simulations (Fig 10B, 10C and 10D, left), we also examined the case in which TD-RPE is calculated according to another RL algorithm called SARSA [30] as follows:

δ(t)=R(S(t))+γQ(A(t))−Q(A(t−1)), (2)

where *A*(*t*) represents the action taken at time step *t*.

At each time step other than the beginning of a trial, the learned value of *A*(*t* − 1) was assumed to be updated as follows:

$$Q{\left(A\left(t-1\right)\right)}_{\text{new}}=Q{\left(A\left(t-1\right)\right)}_{\text{old}}+\alpha \delta \left(t\right),$$ (3)

where *α* is the learning rate (0 ≤ *α* ≤ 1). It was further assumed that the learned value of arbitrary action *A* decays at every time step as follows:

$$Q{\left(A\right)}_{\text{new}}=\left(1-\varphi \right)Q{\left(A\right)}_{\text{old}},$$ (4)

where *φ* (0 ≤ *φ* ≤ 1) is a parameter referred to as the decay rate: *φ* = 0 corresponds to the case without value-decay. This sort of value-decay was introduced in [43] to account for the ramp-like activity of DA neurons reported in [21], and was analyzed in [23]. In the present study, the decay rate *φ* was varied from 0 to 0.02 by 0.002, unless otherwise described. Note that because (1 − *φ*) is multiplied at every time step, even if *φ* is very close to 0, significant decay can occur during a trial. For example, when the decay rate *φ* is 0.01, the action values decline to at least (1–0.01)^7^ (≈ 0.932)-fold of the original values during a trial. It should also be noted that the value-decay defined as above is fundamentally different from the decay of eligibility trace, which is a popular notion in the RL theory [25]: in terms of the eligibility trace, we assumed that only the value of the immediately preceding action (Q(*A*(*t* − 1))) is eligible for RPE-dependent update (Eq (3)), corresponding to the TD(0) algorithm.

At each time step other than when the goal was reached, action ‘Go’ or ‘Stay’ was assumed to be selected according to the following probabilities:

$$P\left(A_{Go}\right)=\frac{\text{exp}\left(\beta Q\left(A_{Go}\right)\right)}{\text{exp}\left(\beta Q\left(A_{Go}\right)\right)+\text{exp}\left(\beta Q\left(A_{Stay}\right)\right)}$$ (5)

$$P\left(A_{Stay}\right)=\frac{\text{exp}\left(\beta Q\left(A_{Stay}\right)\right)}{\text{exp}\left(\beta Q\left(A_{Go}\right)\right)+\text{exp}\left(\beta Q\left(A_{Stay}\right)\right)}\;,$$ (6)

where *β* is a parameter called the inverse temperature, which represents the sharpness of the soft-max selection [134].

A trial ended when subject reached the goal and got the reward. Subsequently the subject was assumed to be (automatically) returned to the start (*S*_1_), and the next trial began. The learning rate *α*, the inverse temperature *β*, and the time discount factor *γ* were set to *α* = 0.5, *β* = 5, and *γ* = 1 unless otherwise described. Initial values of all the action values were set to 0. The amount of reward obtained at the goal, *r*, was set to 1 in most simulations and analyses, but we also examined the cases with *r* = 0.5, 0.75, 1.25, or 1.5 (Fig 11). The magnitude of rewards can in reality vary even more drastically. However, it has been shown [135] that the gain of DA neuron's response adaptively changes according to actual reward sizes. It could thus be possible to assume that *r* does not vary too drastically by virtue of such adaptive mechanisms. In a separate set of simulations (Fig 13), in order to examine the robustness of the effect of the value-decay to perturbations in reward environments, we assumed that there is also small reward, with size *x*, at *S*_4_, which is given whenever subject is located at *S*_4_ (i.e., repeatedly at every time step if subject stays at *S*_4_).

In order to examine the dependence of the effect of the value-decay on the number of states from the start to the goal, we also conducted simulations for models that were modified to have 4 or 10 states, including the start and the goal, instead of 7 states in the original model (Fig 12A and 12B). We also examined the case where the subject is allowed to take not only ‘Go’ or ‘Stay’ but also ‘Back’ action at *S*_*i*_ (*i* = 2, 3, …, 6) (for this, we again assumed 7 states), which causes a backward transition to *S*_*i*−1_. In this case (Fig 12C), selection of ‘Go’, ‘Stay’, and ‘Back’ at *S*_*i*_ (*i* = 2, 3, …, 6) was assumed to be according to the probabilities: *P*(A_*_) = exp(*βQ*(A_*_))/*Sum*, where A_*_ was either *A*_*Go*_, *A*_*Stay*_, or *A*_*Back*_, and *Sum* was exp(*βQ*(*A*_*Go*_)) + exp(*βQ*(*A*_*Stay*_)) + exp(*βQ*(*A*_*Back*_)). Initial values of all the action values, including the values of ‘Back’ actions, were set to 0.

Further, in a separate set of simulations (Fig 9), we considered a different model in which selection of ‘Go’ or ‘Stay’ is based on the state values rather than the action values (‘Back’ was not considered in this model). Specifically, in this model, RPE is calculated as:

δ(t)=R(S(t))+γV(S(t+1))−V(S(t)), (7)

where *V*(*S*(*t*)) represents the state value of *S*(*t*); if *S*(*t*) = *S*_7_, *V*(*S*(*t* + 1)) is assumed to be 0. The state values are updated as follows:

$$V{\left(S\left(t\right)\right)}_{\text{new}}=V{\left(S\left(t\right)\right)}_{\text{old}}+\alpha \delta \left(t\right).$$ (8)

The learned value of arbitrary state *S* was assumed to decay at every time step as follows:

$$V{\left(S\right)}_{\text{new}}=\left(1-\varphi \right)V{\left(S\right)}_{\text{old}}.$$ (9)

‘Go’ is selected at *S*_*i*_ (*i* = 2, 3, …, 6) with the probability exp(*βV*(*S*_*i+*1_))/{exp(*βV*(*S*_*i*_)) + exp(*βV*(*S*_*i+*1_))}, and ‘Stay’ is selected otherwise. The parameters were set to *α* = 0.5, *β* = 5, *γ* = 1, and *φ* = 0.01, and initial values of all the state values were set to 0.

For each condition with different parameter values or model architectures, 20 simulations of 500 trials with different series of pseudorandom numbers were performed, unless otherwise described. The particular number 500 was chosen because it was considered to be largely in the range of the number of trials used in experiments: e.g., in [6], rats completed ~15 or more sessions with each session containing 40 trials. 20 simulations could be interpreted to represent 20 subjects. In the figures showing the number of time steps needed for goal-reaching, we presented the mean ± standard error (SE) of the 20 simulations except for Fig 13E, where the mean ± SE for the simulation runs completing 500 trials (which could be less than 20 for several conditions) were presented. We also presented the theoretical minimum (in the model with 7 states, it is 7, including the steps at the start and the goal) and the chance level, which is calculated (in the model with 7 states) as:

$$7+\left\{1\cdot h\left(6,1\right)\cdot \frac{1}{2}+2\cdot h\left(6,2\right)\cdot {\left(\frac{1}{2}\right)}^{2}+3\cdot h\left(6,3\right)\cdot {\left(\frac{1}{2}\right)}^{3}+\cdots \right\}\cdot {\left(\frac{1}{2}\right)}^{6}=13,$$ (10)

where *h*(6, *k*) represents the number of ways for a repeated (overlapping) combination of *k* out of 6 and is calculated as *h*(6, *k*) = (*k* + 5)!(k! · 5!). Simulations were performed using MATLAB (MathWorks Inc.). Program files to run simulations and make figures are available from ModelDB (https://senselab.med.yale.edu/modeldb/showModel.cshtml?model=195890) after the publication of this article.

### Modeling blockade of DA signaling

To simulate post-training blockade of DA signaling, we replaced *δ*(*t*) in Eq (3) with 0 (complete blockade) or *δ*(*t*)/4 (partial blockade) after 250 trials (Figs 2C, 4 and 6D) or 500 trials (Figs 5 and 14) were completed. *δ*(*t*) was non-negative in those simulations because of the structure of the simulated tasks and the assumed Q-leaning-type calculation of RPE, and so the replacement of *δ*(*t*) with 0 or *δ*(*t*)/4 corresponded to that the size of an increment of action values according to non-negative RPE was reduced to zero or to a quarter of the original size. Notably, at the cellular/synaptic level, DA is known to have two major functions: (i) induce/modulate plasticity of corticostriatal synapses, and (ii) modulate responsiveness of striatal neurons [136]. Function (i) has been suggested to implement RPE-dependent update of learned values (Eq (3)) (e.g., [18]), and in the present work we incorporated the effect of DA blockade on this function into the model as described above, although function (ii) can also affect reaction time and valuation (e.g., [43]) and assuming both of (i) and (ii) might be necessary to account for a wider range of phenomena caused by DA manipulations, in particular, changes in the speed or response time of a single rapid movement (e.g., [137, 138]) rather than (or in addition to) of a series of actions.

### Reduced dynamical system model of ‘Go’ or ‘Stay’ selection, and bifurcation analysis

In order to obtain qualitative understandings of how the value-decay affects the time evolution and steady-state of action values, beyond observations of simulation results, we reduced the original model (Fig 1) to a simpler model through approximations, and conducted bifurcation analysis. Specifically, we considered a reduced continuous-time dynamical system model that approximately describes the time evolution of the values of ‘Stay’ and ‘Go’ at the state preceding the goal (i.e., *A*_11_ (‘Stay’) and *A*_12_ (‘Go’) at *S*_6_ in Fig 1). The reduced model is as follows:

$$\frac{dq\left(A_{11}\right)}{dt}=y\alpha {\tilde{\delta }}_{A_{11}}-\psi q\left(A_{11}\right)$$ (11)

$$\frac{dq\left(A_{12}\right)}{dt}=\alpha {\tilde{\delta }}_{A_{12}}-\psi q\left(A_{12}\right),$$ (12)

where *q*(*A*_11_) and *q*(*A*_12_) are the continuous-time variables that approximately represent the action values of *A*_11_ (‘Stay’) and *A*_12_ (‘Go’), respectively. *y* approximately represents the expected value of the number of repetitions of *A*_11_ (‘Stay’) choice (i.e., how many time steps subject chooses *A*_11_ (‘Stay’) at *S*_6_) in a single trial, and it is calculated as:

$$y=1\cdot p\left(A_{11}\right)\cdot \left(1-p\left(A_{11}\right)\right)+2\cdot p{\left(A_{11}\right)}^{2}\cdot \left(1-p\left(A_{11}\right)\right)+\cdots =\frac{p\left(A_{11}\right)}{1-p\left(A_{11}\right)},$$ (13)

where *p*(*A*_11_) represents the probability that *A*_11_ is chosen out of *A*_11_ and *A*_12_ according to Eq (6) when the values of *A*_11_ and *A*_12_ are *q*(*A*_11_) and *q*(*A*_12_), respectively:

$$p\left(A_{11}\right)=\frac{\text{exp}\left(\beta q\left(A_{11}\right)\right)}{\text{exp}\left(\beta q\left(A_{11}\right)\right)+\text{exp}\left(\beta q\left(A_{12}\right)\right)},$$ (14)

and substituting Eq (14) into Eq (13) results in:

$$y=\text{exp}\left(\beta \left(q\left(A_{11}\right)-q\left(A_{12}\right)\right)\right).$$ (15)

${\tilde{\delta }}_{A_{11}}$ and ${\tilde{\delta }}_{A_{12}}$ represent TD-RPE generated when *A*_11_ or *A*_12_ with the value *q*(*A*_11_) or *q*(*A*_12_) is chosen, respectively:

$${\tilde{\delta }}_{A_{11}}=\gamma \text{max}\left\{q\left(A_{11}\right),q\left(A_{12}\right)\right\}-q\left(A_{11}\right)$$ (16)

$${\tilde{\delta }}_{A_{12}}=r-q\left(A_{12}\right),$$ (17)

where *r* is the reward amount (= 1). *ψ* is a parameter representing the degree of the value-decay in a trial, which roughly corresponds to the decay rate *φ* in the original model multiplied by the number of time steps needed for goal-reaching. Notably, the reduced model is a continuous-time approximation of an algorithm in which update and decay of learned values occur once per every trial in a batch-wise manner whereas the original model is described as an online algorithm where update and value-decay occur at every time step; this difference is contained in our expression "approximate" referring to the reduced model. We analyzed the two-dimensional dynamics of *q*(*A*_11_) and *q*(*A*_12_) (Eqs (11) and (12)) under the assumption that *q*(*A*_11_) ≤ *q*(*A*_12_) (i.e., max{*q*(*A*_11_), *q*(*A*_12_)} = *q*(*A*_12_) in Eq (16)). More specifically, we numerically solved the equations $\frac{dq\left(A_{11}\right)}{dt}=0$ and $\frac{dq\left(A_{12}\right)}{dt}=0$ to draw the nullclines (Fig 7E), and also numerically found the equilibriums and examined their stabilities to draw the bifurcation diagram (Fig 7B) and calculate *p*(*A*_11_) and *p*(*A*_12_) (Fig 7C) by using MATLAB. The result of the bifurcation analysis in the case with *α* = 0.5, *β* = 5, and *γ* = 1 (Fig 7B) was further confirmed by using XPP-Aut (http://www.math.pitt.edu/~bard/xpp/xpp.html).

### Simulation of a cost-benefit decision making task in a T-maze

We simulated an experiment examining the effects of DA depletion in the nucleus accumbens in a T-maze task reported in [24]. There were two conditions in the task. In the first condition, there was small reward in one of the two arms of the T-maze whereas there was large reward accompanied with high cost (physical barrier preceding the reward) in the other arm. In the second condition, the two arms contained small and large rewards as before, but neither was accompanied with high cost. We simulated this experiment by representing the high cost as an extra state preceding the reward. Specifically, we assumed a state-action diagram as shown in Fig 5A and 5E (right panels). There were two action candidates, ‘Go’ and ‘Stay’, at every state, except for the state at the T-junction (State 4) and the state at the trial end, which was reached if ‘Go’ was chosen at State 7 or 8. In State 4, there were three action candidates, ‘Choose, and Go to, one of the arm (Arm 1)’, ‘Choose, and Go to, the other arm (Arm 2)’, and ‘Stay’. In the state at the trial end (State 9, which is not depicted in Fig 5A and 5E), there was no action candidate, and subject was assumed to be automatically moved to the start state (State 1) at the next time step. In the first condition of the simulated task (Fig 5A), small reward (size 0.5) was given when subject reached State 6 for the first time (i.e., only once in a trial), whereas large reward (size 1) was given when subject reached State 7 for the first time. One extra state, i.e., State 5, preceding the state associated with large reward (State 7) was assumed to represent high cost accompanied with the large reward. In the second condition (Fig 5E), small (size 0.5) or large (size 1) reward was given when subject reached State 6 or State 5, respectively, for the first time, representing that neither reward was accompanied with high cost. Calculation of Q-learning-type RPE and RPE-dependent update of action values were assumed in the same manner as before, with the parameters *α* = 0.5, *β* = 5, and *γ* = 1. The value-decay was also assumed similarly, with the decay rate *φ* = 0.01. Initial values of all the action values were set to 0. 20 simulations of 1000 trials were conducted for each condition, and post-training DA depletion was simulated in such a way that the size of RPE-dependent increment of action values was reduced to a quarter of the original size after 500 trials were completed.

### Elaborated model aiming at reproducing velocity profiles in a T-maze

By modifying the original model described above, we developed an elaborated model of self-paced spatial movement, and simulated the cost-benefit decision making task in a T-maze mentioned above. In this elaborated model, the exact one-to-one correspondence between the subject's physical location and the internal state assumed in the original model was changed into a loose coupling, in which each state corresponds to a range of physical locations (Fig 14A). Also, ‘Stay’ action in the original model was replaced with ‘Slow’ action unless there is a physical constraint (i.e., the start, the T-junction, or the end). Specifically, it was assumed that, at each time step *t*, subject at a given location chooses either ‘Go’ or ‘Slow’, except that the subject is at the start, T-junction, or the reward location (in the ends of the T-maze). By selecting ‘Go’, subject moves straightforward for a time step with the "velocity" 1, meaning that the subject's physical location is displaced by 1, or moves to the T-junction or the reward location when it is within 1 from the current location. By selecting ‘Slow’, subject moves straightforward for a time step with the "velocity" halved, meaning that the subject's physical location is displaced by the half of the displacement during the previous time interval (between *t* − 1 and *t*), or moves to the T-junction or the reward location when it is within the calculated displacement from the current location. In these ways, the "velocity" in this model was defined as the displacement in a time step. At the start (State 1), subject was assumed to take ‘Go’ or ‘Stay’ as in the original model (because at the start, the previous "velocity" was not defined). At the T-junction, subject was assumed to take ‘Choose, and Go to, one of the arm (Arm 1)’, ‘Choose, and Go to, the other arm (Arm 2)’, or ‘Stay’. By selecting ‘Choose, and Go to, Arm 1 or 2’, the subject's physical location is displaced by 1 on the selected arm. By selecting ‘Stay’, subject stays at the same place (T-junction). At the reward location, subject was assumed to take the consummatory action for a time step (indicated by the double-lined arrows in Fig 14B and 14F), and proceed to the end state. Calculation of Q-learning-type TD-RPE, update of action values, and the value-decay were assumed in the same manner as in the original model.
